# Identification and validation of prognostic genes related to glycolysis and M2 macrophage in hepatocellular carcinoma: an integrated analysis of bulk RNA sequencing and single-cell RNA sequencing

**DOI:** 10.3389/fimmu.2026.1710411

**Published:** 2026-02-12

**Authors:** Anqi Wang, Lina You, Ming Zuo, Zhanao He, Hong Yang, Wukui Huang

**Affiliations:** 1 Third Clinical Medical College of Xinjiang Medical University (The Affiliated Tumor Hospital of Xinjiang Medical University), Urumqi, Xinjiang, China; 2 Department of Traditional Chinese Medicine Oncology, The Affiliated Tumor Hospital of Xinjiang Medical University, Urumqi, Xinjiang, China; 3 Department of Medical, The Affiliated Tumor Hospital of Xinjiang Medical University, Urumqi, Xinjiang, China; 4 Department of Interventional Diagnosis and Treatment, The Affiliated Tumor Hospital of Xinjiang Medical University, Urumqi, Xinjiang, China

**Keywords:** glycolysis, hepatocellular carcinoma, immune infiltration, M2 macrophage, prognostic genes, single-cell RNA sequencing

## Abstract

**Background:**

The role of the crosstalk between glycolysis and M2 macrophages in hepatocellular carcinoma (HCC) progression remains incompletely understood. This study aimed to identify prognostic genes linked to both glycolysis and M2 macrophages in HCC and to elucidate their mechanistic underpinnings.

**Methods:**

Single-cell RNA sequencing (scRNA-seq) and transcriptomic data (TCGA-LIHC) were obtained from public databases. M2 macrophage-related genes (MRGs) were integrated with differentially expressed genes (DEGs1) from immune infiltration analysis and macrophage polarization-related genes (MPRGs). Candidate genes were identified through the intersection of glycolysis-related genes (GRGs), MRGs, and HCC-control DEGs (DEGs2). Prognostic genes were selected *via* regression analysis for the development of a risk model. Subsequent analyses included nomogram development, functional enrichment, immune characterization, and drug sensitivity assessment. Single-cell analysis highlighted key cell populations and prognostic gene expression profiles in HCC. RT-qPCR was performed to validate prognostic gene expression levels.

**Results:**

Fifty-two candidate genes were identified from the intersection of GRGs, MRGs, and DEGs2. Eight genes—PFKFB4, ADH4, ADH1C, ME1, FOXK1, PFKP, ARL2, and TKTL1—were selected as prognostic genes. ADH4 and ADH1C exhibited significantly higher expression in the low-risk group, whereas the other genes were elevated in the high-risk group. A more accurate risk model and nomogram were developed. Further analyses indicated that the prognostic genes might contribute to HCC progression through pathways such as drug metabolism (cytochrome P450), immune cell infiltration (naive B cells and M2 macrophages), immune escape, and drug sensitivity (e.g., A.770041), potentially influencing cellular interactions and differentiation states of hepatocytes and M2 macrophages. RT-qPCR confirmed that PFKFB4, FOXK1, and TKTL1 were upregulated in HCC, while ADH4 and ADH1C were downregulated.

**Conclusion:**

Eight prognostic genes were identified, and a risk model was established, providing valuable insights for clinical prognostic prediction and immunotherapy in HCC.

## 1 Introduction

Hepatocellular carcinoma (HCC) is the most common form of primary liver cancer, accounting for more than 90% of liver cancer cases (1). As an exceptionally aggressive tumor, it represents a major global health challenge, with the sixth highest incidence and the third highest mortality rate among all cancers worldwide (2). Common screening methods for HCC include serum alpha-fetoprotein (AFP) testing, ultrasonography, and computed tomography (CT) scans. However, most patients are diagnosed at advanced stages, leading to a poor prognosis, with a 5-year survival rate of less than 20%. Consequently, identifying prognostic genes for HCC, developing prognostic models, and elucidating the mechanisms underlying these genes are critical for advancing both the understanding of liver cancer pathogenesis and the development of immunotherapies.

Aerobic glycolysis, also known as the Warburg effect, plays a vital role in HCC development and progression by modulating various pathways, including tumor cell proliferation, invasion, metastasis (3), apoptosis, cell cycle progression, immune evasion, and angiogenesis. Numerous immunosuppressive cell populations, such as regulatory T cells (Tregs) and myeloid-derived suppressor cells, are integral to tumor progression within the tumor microenvironment (TME). M2-polarized macrophages, in particular, stand out due to their functional plasticity and close interaction with tumor cells. These macrophages contribute to an immunosuppressive environment by secreting cytokines like IL-10 and TGF-β, while simultaneously promoting angiogenesis, tissue remodeling, and extracellular matrix reorganization, directly supporting tumor growth and metastasis (4, 5). Accumulating evidence highlights that metabolic reprogramming in tumor cells, especially enhanced glycolysis, significantly shapes the TME. Glycolysis-derived metabolites, such as lactate, can induce macrophage polarization toward the M2 phenotype, establishing a positive feedback loop that accelerates tumor progression (6, 7). Therefore, M2 macrophages play a pivotal role in linking tumor glycolytic metabolism to the immunosuppressive TME. This study focuses on the interaction between M2 macrophages and glycolysis-related metabolic alterations, exploring their prognostic relevance in. Although conventional biomarkers, such as serum AFP, glypican-3 (GPC3), and des-γ-carboxy prothrombin (DCP), are commonly used in the clinical diagnosis of HCC, their sensitivity and specificity are limited, particularly for early detection and prediction of therapeutic response (7). Recent advancements in liquid biopsy and multi-omics technologies have catalyzed a new phase of biomarker discovery in HCC. Aerobic glycolysis and M2-polarized tumor-associated macrophages are two interrelated hallmarks of HCC progression. However, gene signatures resulting from the interplay between tumor glycolytic metabolism and M2 macrophage polarization have yet to be systematically characterized. Investigating these integrated prognostic gene signatures could offer a novel framework for risk stratification and personalized management of patients with HCC.

Single-cell RNA sequencing (scRNA-seq) allows for high-resolution analysis of cellular heterogeneity and intercellular communication within the TME, providing a powerful tool to investigate metabolic–immune interactions at the cellular level (8, 9). In the present study, scRNA-seq data were utilized to examine the spatial distribution and functional association between glycolysis-related genes (GRGs) and M2-polarized macrophages in HCC. By integrating single-cell transcriptomic evidence, the study aimed to more accurately elucidate the cooperative mechanisms linking tumor glycolytic metabolism with M2 macrophage–mediated immunosuppression.

Although previous studies have separately explored the roles of glycolysis and M2-polarized macrophages in HCC, there has been limited investigation into their interactive contributions to HCC progression and prognosis. This study integrated bulk transcriptomic data with scRNA-seq to systematically identify and preliminarily validate prognostic genes associated with both aerobic glycolysis and M2 macrophages. Using this metabolic–immune interaction axis, a prognostic risk model for survival prediction in HCC was developed. Additionally, single-cell analyses were employed to investigate the expression dynamics of these prognostic genes in hepatocytes and M2 macrophages, as well as their potential cell–cell communication patterns. This integrative analytical approach not only offers a novel prognostic gene signature but also provides mechanistic insights into the interplay between metabolic reprogramming and immune microenvironment remodeling in HCC, laying the foundation for immunometabolic stratification and therapeutic strategies for immunometabolic stratification and therapeutic strategies.

## 2 Materials and methods

### 2.1 Data source

Training data comprised 368 HCC samples and 50 control samples from The Cancer Genome Atlas (TCGA)-LIHC dataset (https://portal.gdc.cancer.gov/). Validation was conducted using 218 HCC samples from the International Cancer Genome Consortium (ICGC) database (https://dcc.icgc.org/). For single-cell analysis, the GSE149614 dataset (GPL24676) containing 10 HCC and 8 control samples from the Gene Expression Omnibus (GEO) database (https://www.ncbi.nlm.nih.gov/geo/) was utilized. A total of 341 GRGs and 1980 macrophage polarization-related genes (MPRGs) were retrieved from the MSigDB database (https://www.gsea-msigdb.org/gsea/msigdb) (10, 11).

### 2.2 Functional pathways and protein interaction analyses of candidate genes

Based on the M2 macrophage infiltration scores estimated by the CIBERSORT algorithm (12) (v1.03), HCC samples in the training cohort were categorized into high- and low-infiltration groups for further analysis. Differential gene expression analysis between these stratified groups was conducted using the DESeq2 package (13) (v1.40.2) to identify differentially expressed genes (DEGs1) with criteria |log_2_ fold change (FC)| > 1 and adjusted P-value< 0.05. Gene expression patterns were visualized with volcano plots (created using ggplot2 (14) v3.5.0) and hierarchical heatmaps (generated using pheatmap (15) v1.0.12). To identify M2 macrophage-related genes (MRGs), DEGs1 were integrated with a predefined list of MPRGs after removing duplicates. A parallel differential expression analysis between HCC and control samples (using identical DESeq2 parameters) generated a second set of DEGs (DEGs2), which were also visualized using the same bioinformatics pipelines. The core analysis pipeline combined three gene sets: GRGs, MRGs, and DEGs2. A Venn diagram analysis (VennDiagram package (16) v1.7.1) identified overlapping genes across these datasets, which were designated as glycolysis- and M2 macrophage-related candidate genes (GMRGs). Functional characterization of GMRGs was performed using clusterProfiler (v4.2.2 (17)) for Gene Ontology (GO) biological process enrichment and Kyoto Encyclopedia of Genes and Genomes (KEGG) pathway analysis (significance threshold: P< 0.05). Protein-protein interaction (PPI) networks were constructed using the STRING database (confidence threshold: 0.4) and visualized through Cytoscape (18) (v3.1.1) to explore potential molecular interactions among candidate genes.

### 2.3 Identification of prognostic genes

To assess the survival prediction potential, candidate genes underwent univariate Cox analysis within the training set (HR ≠ 1, P< 0.01), followed by proportional hazards assumption testing using the cox.zph function from the survival package (19) (v3.5-3). A P-value > 0.05 suggests no violation of the proportional hazards assumption. Genes meeting these criteria were considered as candidate prognostic genes, which were visualized using the forestplot package (20) (v2.0.1) and further analyzed *via* least absolute shrinkage and selection operator (LASSO) regression to identify final prognostic genes. LASSO analysis was performed using the glmnet package (v 4.1-4) (21), with a random seed set to 2 and 10-fold cross-validation.

### 2.4 Construction and validation of risk model

Based on the prognostic genes identified in the training set, a risk model was constructed using the following formula (where coef and expr denote the risk coefficient and expression level of each gene, respectively):


$$Risk\;score=\sum_{i=1}^{n}coef\;\left(gene_{i}\right)\times \exp r\;(gene_{i})$$

HCC samples were categorized into high- and low-risk groups using the median risk score as the cutoff threshold. Risk score distribution and survival status were visualized using the survminer (22) (v0.4.9) and ggrisk (23) (v1.3) packages. Prognostic gene expression patterns across risk groups were visualized *via* heatmaps. Kaplan-Meier (KM) survival curves comparing the two risk groups were generated using the survminer package (v0.4.9), with survival differences assessed by log-rank testing (P< 0.05). Receiver operating characteristic (ROC) curves at 1-, 3-, and 5-year time points were constructed using survivalROC (24) (v1.0.3) and ggplot2 (v3.5.0) packages to calculate area under the curve (AUC) values. The model’s accuracy and generalizability were further evaluated using the validation set.

### 2.5 Independent prognostic analysis and nomogram establishment

Risk score variations across clinical parameters (age, gender, grade, T/N/M stages) were analyzed using Wilcoxon rank sum tests (P< 0.05) to explore associations with patient characteristics. Sequential univariate and multivariate Cox regression analyses identified independent prognostic factors (HR ≠ 1, P< 0.05). A predictive nomogram was developed using the rms package (25) (v6.7-0) to estimate 1-, 3-, and 5-year survival probabilities based on significant clinical variables and risk scores. Model validation was performed using calibration plots and ROC curve analysis. Calibration curve slopes approximating unity demonstrated good agreement between predicted and observed outcomes, while AUC values greater than 0.7 confirmed robust discriminative performance across all time points.

### 2.6 Differential functional pathways analysis

To investigate relevant pathways and biological mechanisms between the two risk groups, differential analysis within the training set was conducted using the DESeq2 package (v1.40.2). The log_2_FC values from this analysis were sorted from largest to smallest and used as the sorting criterion. Gene set enrichment analysis (GSEA) was conducted with the clusterProfiler package (v4.2.2) (P.adjust< 0.05, seed = 1). The reference gene set used was “c2.cp.kegg.v7.5.1.symbols.gmt,” obtained *via* the msigdbr package (26) (v7.5.1).

### 2.7 Immune microenvironment-related and drug sensitivity analyses

Immune microenvironment differences between the risk groups were analyzed within the training set. The CIBERSORT algorithm (v1.03) was used to quantify the relative percentages of 22 immune infiltrating cell types, with significant differences identified through Wilcoxon rank sum testing (P.adjust< 0.05, false discovery rate [FDR] correction applied). The ESTIMATE algorithm (27) (v1.0.13) was utilized to calculate stromal scores, immune scores, ESTIMATE scores, and tumor purity for both risk groups. Immune exclusion, immune dysfunction, and tumor immune dysfunction and exclusion (TIDE) scores were calculated using the TIDE official website (http://tide.dfci.harvard.edu/), with between-group differences assessed using Wilcoxon rank sum testing (P< 0.05). Expression levels of 48 immune checkpoint-related genes from the literature (28) were compared between risk groups using Wilcoxon rank sum analysis (P.adjust< 0.05, FDR correction applied). Tumor mutational burden (TMB) data for patients with HCC in the training set were used to conduct somatic mutation analysis and visualization through the maftools package (v2.20.0) (https://github.com/poisonalien/maftools). TMB differences between risk groups were evaluated using Wilcoxon rank sum testing (P< 0.05). Drug sensitivity analysis was conducted using 138 chemotherapy agents from the Genomics of Drug Sensitivity in Cancer (GDSC) database (https://www.cancerrxgene.org/). The pRRophetic package (29) (v0.5, seed = 123456) was used to calculate IC_50_ values for HCC samples, with inter-group IC_50_ differences assessed through Wilcoxon rank sum testing (P.adjust< 0.05, FDR correction applied).

### 2.8 Gene set variation and cell-cell communication analysis on candidate key cells

To investigate prognostic gene expression at the single-cell level, comprehensive analyses were conducted using the scRNA-seq dataset. Quality control was performed with the Seurat package (30) (v5.1.0), filtering cells with gene counts between 200 and 6,000 and molecules< 20,000. The PercentageFeatureSet function was used to calculate mitochondrial gene percentages, selecting cells with< 5% mitochondrial genes for analysis. Data standardization *via* NormalizeData and FindVariableFeatures functions identified the 2,000 most variable genes. Principal component analysis (PCA), performed with RunPCA, JackStrawPlot, and ElbowPlot functions, determined the significant principal components for downstream analysis (P< 0.05). Following PCA dimensionality reduction, unsupervised clustering of filtered cells was conducted using FindNeighbors and FindClusters functions (resolution = 0.3). UMAP clustering revealed distinct cell clusters. Cell annotation was carried out using the singleR package (31) (v2.0.0), with M1 macrophages annotated by CD86, IL1B, and CXCL9 markers, and M2 macrophages by CCL18, CD163, and MRC1 markers. The percentage of each cell type in HCC and control groups was compared using Wilcoxon rank sum testing (P< 0.05) to identify key candidate cells. Gene set variation analysis (GSVA) using the Hallmark gene set from the MSigDB database characterized biological pathways associated with key candidate cells. Intercellular communication between these candidate key cells and other cell types in HCC and control groups was assessed using the cellchat package (32) (v1.5.0).

### 2.9 Cell trajectory analysis on key cells

To identify key cells, prognostic gene expression across annotated cell types was visualized with UMAP plots, and expression differences between HCC (tumor) and control (normal) groups were compared. To further elucidate the mechanisms of prognostic genes within key cells, UMAP dimensionality reduction clustered these cells into distinct subpopulations. Cell trajectory analysis, using the monocle package (v2.26.0) (33), simulated the differentiation of key cells and characterized prognostic gene expression patterns across developmental stages.

### 2.10 Expression level verification of prognostic genes and analysis of protein content

In this study, 5 pathologically confirmed HCC tumor tissue samples and 5 paraneoplastic control tissue samples were collected from the Affiliated Tumor Hospital of Xinjiang Medical University. The study was approved by the Ethics Committee of the Affiliated Tumor Hospital of Xinjiang Medical University (2024BC032), and all participants provided informed consent. Total RNA was extracted from approximately 50 mg of tissue samples using TRIzol reagent (Ambion, USA). RNA integrity and purity were assessed through electrophoresis and NanoPhotometer N50 analysis, respectively. cDNA synthesis was performed using the SureScript First-strand cDNA synthesis kit (Servicebio, China). Reverse transcription-quantitative PCR (RT-qPCR) was conducted with Universal Blue SYBR Green qPCR Master Mix (Servicebio, China) on a CFX Connect system (BIO-RAD, USA). Each sample was tested in triplicate. Primer sequences for prognostic genes and GAPDH (internal control) are listed in **Supplementary Table S1**, with detailed reaction conditions provided in **Supplementary Tables S2**-**S5**. Relative expression levels were calculated using the 2^-ΔΔCт^ method. Statistical analysis was conducted using t-tests (P< 0.05), and data visualization was performed using GraphPad Prism software (34) (v8.0). Additionally, protein expression levels of prognostic genes were analyzed using the Human Protein Atlas (HPA) database (https://www.proteinatlas.org/).

### 2.11 Statistical analysis

Bioinformatics analyses were conducted in R (v4.2.2). Group comparisons were performed using Wilcoxon rank sum and t-tests (P< 0.05). In figures, significance levels were indicated as follows: ns for P > 0.05, * for P< 0.05, ** for 0.001< P< 0.01, *** for 0.0001< P< 0.001, and **** for P< 0.0001. The complete analysis process is illustrated in **Supplementary Figure 1**.

## 3 Results

### 3.1 Acquisition and related functional pathways of candidate genes associated with glycolysis and M2 macrophage

**Figure 1:**
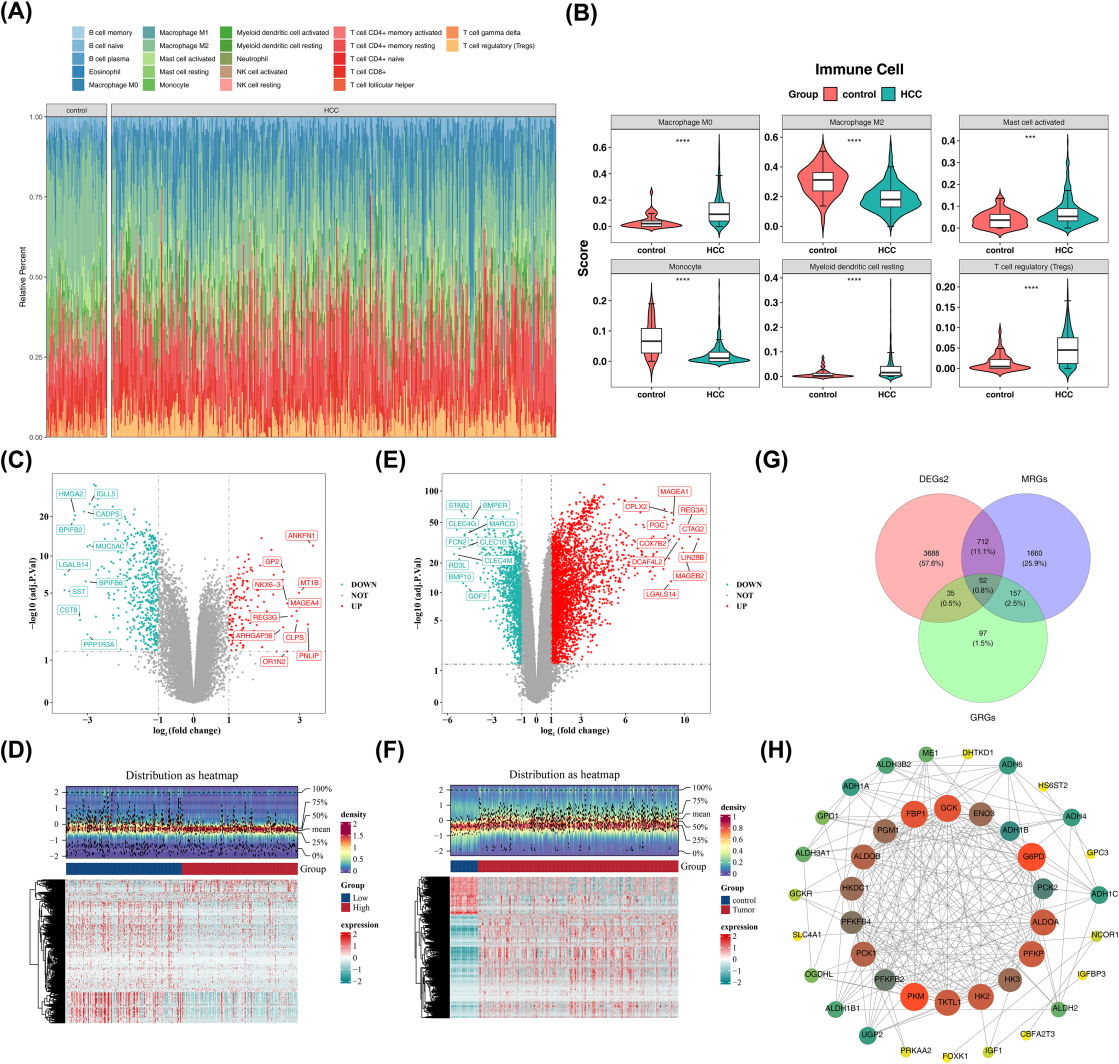
Immune cell infiltration and differential gene expression analysis in HCC. **(A)** Comparison of the relative percentages of 22 immune cell types between HCC tumor samples and control samples.**(B)** Boxline plots revealed the infiltration differences of 22 immune cell types between HCC and control samplers (P<0.05). *** represents P<0.001, and **** represents P<0.0001. **(C, D)** Volcano plot **(C)** and heatmap **(D)** of differentially expressed genes (DEGs1) between HCC samples with high versus low M2 macrophage infiltration. Red indicates upregulated gene expression, while blue indicates downregulated gene expression. The darker the color, the higher or lower the expression. **(E, F)** Volcano plot **(E)** and heatmap **(F)** visualization of 4,487 DEGs2 between HCC samples and control samples. **(G)** Venn plot of the interaction of GRGs, MRGs, and DEGs2. **(H)** Protein-protein interaction (PPI) network. Node size indicates the size of the confidence level. Larger nodes indicate greater confidence. Shades of color, from green to yellow to red to reddish-brown, indicate the size of the degree. The darker the color, the larger the degree.

Relative percentages of 22 immune cell types differed between tumor (HCC) and control samples (**Figure 1A**). Four immune cell populations, including M0 macrophages and Tregs, showed significantly higher infiltration in HCC samples, while M2 macrophages and monocytes exhibited greater infiltration in control samples (P< 0.05) (**Figure 1B**). Between the M2 macrophage high−infiltration and low−infiltration groups, differential expression analysis identified 651 DEGs1, including 140 upregulated genes and 511 downregulated genes (**Figures 1C, D**). Subsequently, the 651 DEGs1 were combined with 1,980 MPRGs retrieved from the MSigDB database, and duplicate entries were removed, resulting in a final integrated set of 2,581 MRGs.

Meanwhile, differential expression analysis between all HCC samples and control samples in TCGA−LIHC identified 4,487 DEGs2, among which 3,260 were upregulated and 1,227 were downregulated (**Figures 1E, F**). Finally, by intersecting the 341 GRGs, the aforementioned 2,581 MRGs, and the 4,487 DEGs2, we identified a total of 52 candidate genes that were present in all three sets (**Figure 1G**).

These candidate genes were significantly enriched across 452 GO entries, including 378 biological processes (BPs) such as pyruvate metabolic process, 17 cellular components (CCs) such as mitochondrial matrix, and 57 molecular functions (MFs) such as monosaccharide binding. The top 5 most significant categories are shown in **Supplementary Figure 2A**. Additionally, candidate genes were enriched in 38 pathways, including glycolysis/gluconeogenesis, carbon metabolism, and pyruvate metabolism, with the top 9 significant pathways visualized in **Supplementary Figure 2B**. These results indicate that the candidate genes are primarily associated with these biological functions and pathways, which provide insights into the potential roles of GMRGs in HCC. The PPI network included 41 proteins with 199 interaction pairs, revealing complex gene interconnections and their potential significance in HCC pathogenesis (**Figure 1H**).

### 3.2 Eight prognostic genes were screened out and their expression levels were preliminarily verified

**Figure 2:**
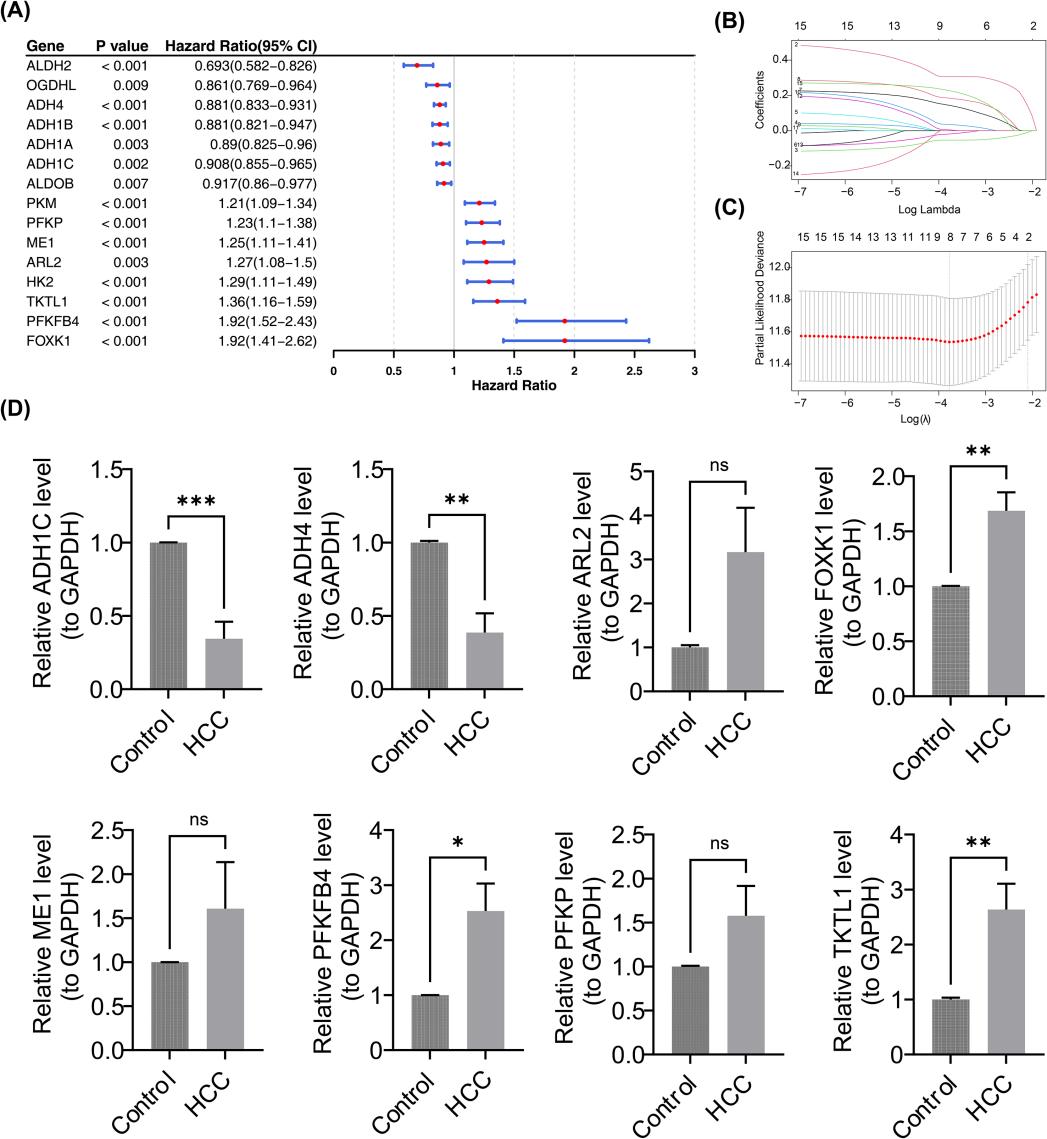
Screening and expression level verification of prognostic genes. **(A)** The forest plot of 15 candidate prognostic genes. Each horizontal line represents the 95% confidence interval of the HR of this gene, and the red dot in the center of the horizontal line is the average value of the HR. **(B, C)** LASSO regression selected eight prognostic genes. The horizontal axes are all log(Lambda), and the vertical axes are the coefficients of genes and the partial likelihood bias respectively. **(D)** Comparison of relative expression levels of prognostic genes between HCC tissues and control tissues. The figure displays the relative mRNA expression levels of genes ADH1C, ADH4, ARL2, FOXK1, ME1, PFKFB4, PFKP, and TKTL1 (normalized to GAPDH as the internal reference). Statistical significance: *P<0.05, **P<0.01, ***P<0.001; “ns” indicates no statistically significant difference. The results show that ADH1C and ADH4 are significantly downregulated in HCC, while FOXK1, PFKFB4, and TKTL1 are significantly upregulated in HCC; no statistically significant differences were observed in the expression of the remaining genes.

Univariate Cox analysis identified 15 candidate prognostic genes (**Figure 2A**). Seven genes (ALDH2, OGDHL, ADH4, ADH1B, ADH1A, ADH1C, ALDOB) were associated with a favorable prognosis (HR< 1, P< 0.05), while eight genes (PKM, PFKP, ME1, ARL2, HK2, TKTL1, PFKFB4, FOXK1) correlated with a poor prognosis (HR > 1, P< 0.05). All 15 candidate prognostic genes passed the proportional hazards assumption test (P > 0.05), indicating that the effects of these genes on patients’ survival risk were constant over time. Thus, the hazard ratios (HRs) estimated based on the Cox regression model were reliable and interpretable. LASSO regression selected eight prognostic genes: PFKFB4, ADH4, ADH1C, ME1, FOXK1, PFKP, ARL2, and TKTL1 (**Figures 2B, C**). The differential expression of these eight prognostic genes between HCC and control samples is shown in **Supplementary Table S6**. To preliminarily verify the expression level of prognostic genes at the experimental level, RT-qPCR was conducted, and the results showed that compared to the control group, the expression of PFKFB4, FOXK1, and TKTL1 was significantly higher in HCC, while ADH4 and ADH1C expression was notably lower (p< 0.05). Although ME1, PFKP, and ARL2 showed a tendency for upregulation in HCC, the differences were not statistically significant, likely due to the small sample size (**Figure 2D**). Furthermore, analysis of the HPA database indicated that the proteins ADH1C, ARL2, FOXK1, and ME1 were more highly expressed in HCC, whereas ADH4, PFKFB4, and PFKP exhibited lower expression levels in HCC (**Supplementary Figure 3**). The discrepancies in the expression trends of mRNA and protein for certain genes (e.g., PFKFB4) may stem from post-transcriptional regulation, differences in translation efficiency, or variations in protein degradation rates. These differential expressions of prognostic genes further emphasize their potential prognostic value in HCC.

### 3.3 Construction of a risk model with superior predictive ability based on eight prognostic genes

**Figure 3:**
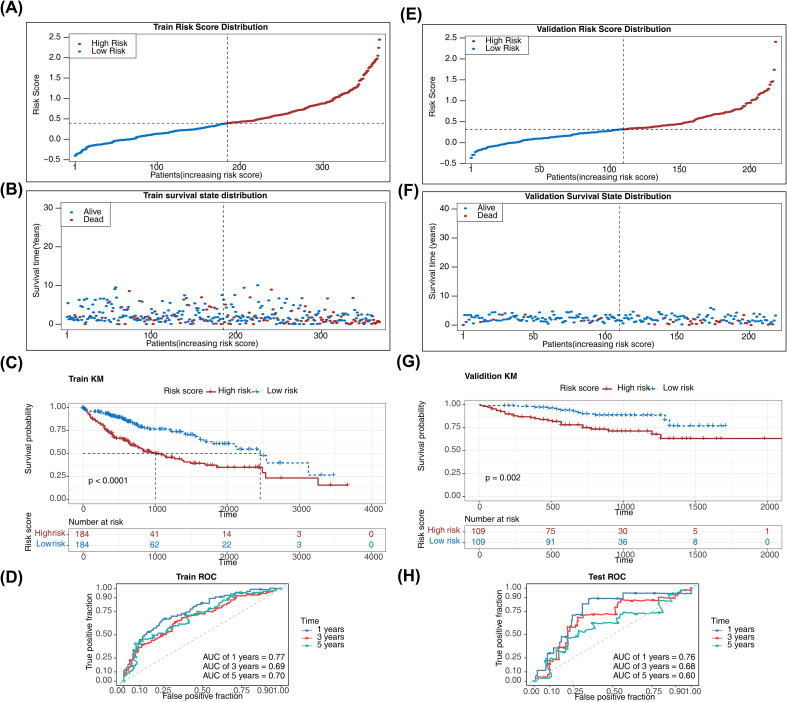
Development and validation of a prognostic risk model for HCC. **(A, B)** Risk score distribution across high-risk and low-risk HCC samples from TCGA--LIHC. **(C)** Kaplan-Meier survival analysis between high-risk and low-risk HCC patients from TCGA--LIHC. The survival rate of patients in the low-risk group was significantly higher than that of patients in the high-risk group (P< 0.0001). **(D)** ROC curves showing AUC values exceeding 0.6 for 1-, 3-, and 5-year survival predictions. **(E, F)** Risk score distribution across high-risk and low-risk HCC samples from validation set. **(G)** Kaplan-Meier survival analysis in the validation set (P = 0.002). **(H)** ROC curves for the validation set.

The risk model was constructed as follows: Risk score = (0.306033748) * PFKFB4 + (-0.053640296) * ADH4 + (-0.018816813) * ADH1C + (0.143684255) * ME1 + (0.184216879) * FOXK1 + (0.034986113) * PFKP + (0.004321028) * ARL2 + (0.226911576) * TKTL1. Risk score distribution indicated higher mortality with increasing scores (**Figures 3A, B**). KM analysis confirmed a significantly higher survival probability in low-risk patients (P< 0.05) (**Figure 3C**). The AUC values for 1-, 3-, and 5-year predictions all exceeded 0.6, demonstrating superior model performance (**Figure 3D**).

The risk model was validated using the validation set. Risk score distribution (**Figure 3E-F**), KM survival analysis (**Figure 3G**), and ROC curves with AUC values exceeding 0.6 for 1-, 3-, and 5-year predictions (**Figure 3H**) confirmed excellent model generalizability. Additionally, C-indices for the risk models in the training set and validation set were 0.627 and 0.644, respectively (**Supplementary Table S7**). These results affirm the effectiveness of the prognostic genes in risk stratification and survival prediction, providing a valuable tool for personalized HCC prognosis in clinical practice.

### 3.4 Establishment of a nomogram integrating clinical characteristics and risk score

**Figure 4:**
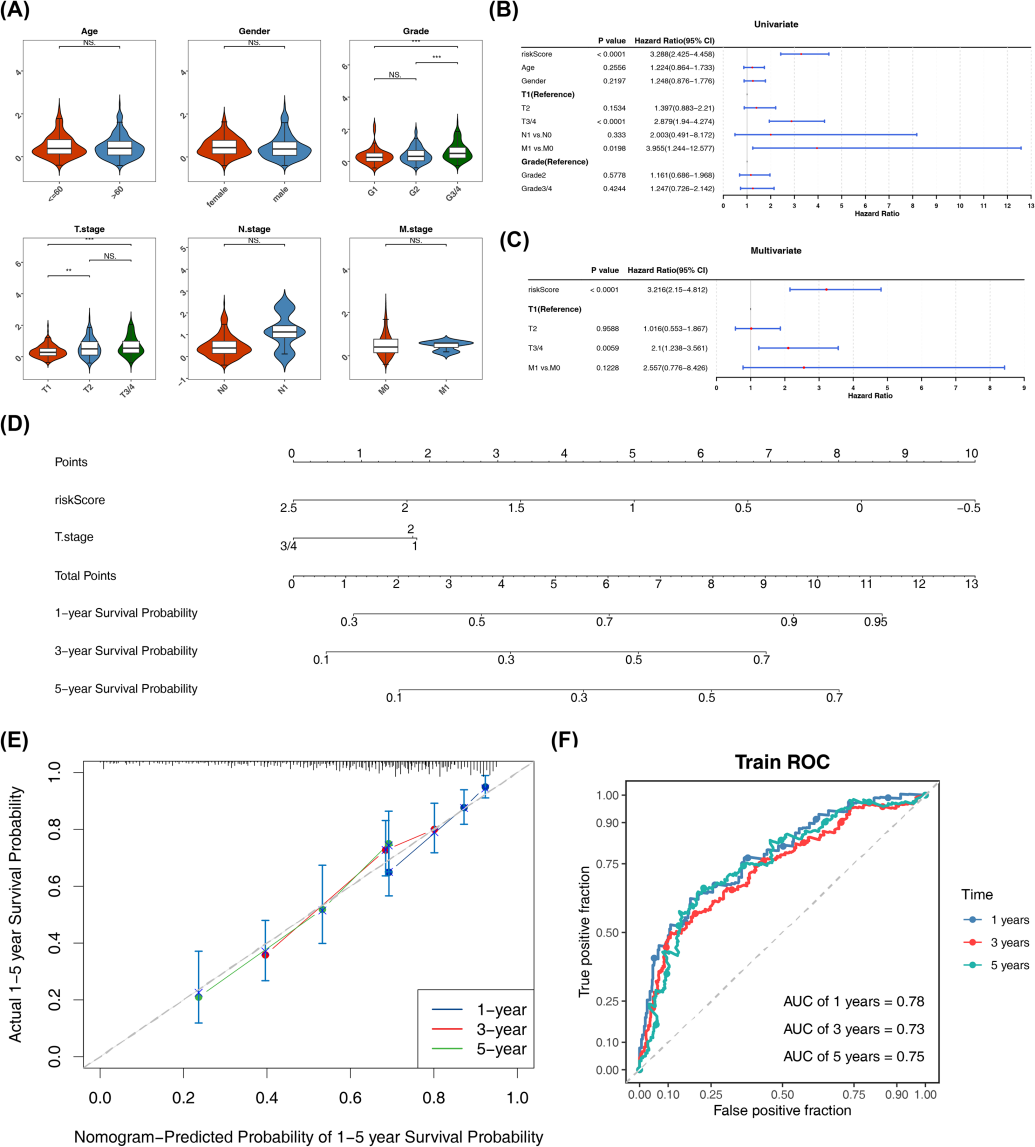
Prognostic analysis and nomogram for HCC. **(A)** Box line plot of the different clinical feature subgroups. ns represents P>0.05, ** represents P<0.01, and *** represents P<0.001 **(B, C)** the forest plots of univariate and multivariate Cox regression analyses. **(D)** A predictive nomogram incorporating risk score and T stage. **(E, F)** Calibration curve **(F)** ROC curves of 1-, 3-, and 5- years.

Risk scores significantly differed across tumor grades and T stages (P< 0.05), confirming their correlation with risk stratification (**Figure 4A**). Univariate Cox regression identified risk score, T stage, and M stage as significant prognostic factors (P< 0.05). Multivariate analysis further validated risk score and T stage as independent predictors (P< 0.05) (**Figures 4B, C**). A predictive nomogram incorporating these variables was constructed (**Figure 4D**), where elevated total scores correlated with improved HCC patients’ survival probability. Model validation showed calibration curve slopes approaching unity and AUC values exceeding 0.7, indicating robust predictive accuracy and clinical applicability for HCC prognostication (**Figures 4E, F**).

### 3.5 Crucial functional pathways altered by risk scores

Pathways such as drug metabolism *via* cytochrome P450, fatty acid metabolism, and glycine, serine, and threonine metabolism were influenced by risk scores (**Supplementary Figure 4**). The role of prognostic genes in HCC is likely multifaceted, involving metabolic reprogramming, signal transduction, and drug resistance. Therefore, a comprehensive investigation of these pathways could potentially lead to improved treatment strategies and prognostic assessments for HCC.

### 3.6 Differential immune microenvironment-related characteristics and drug sensitivities

**Figure 5:**
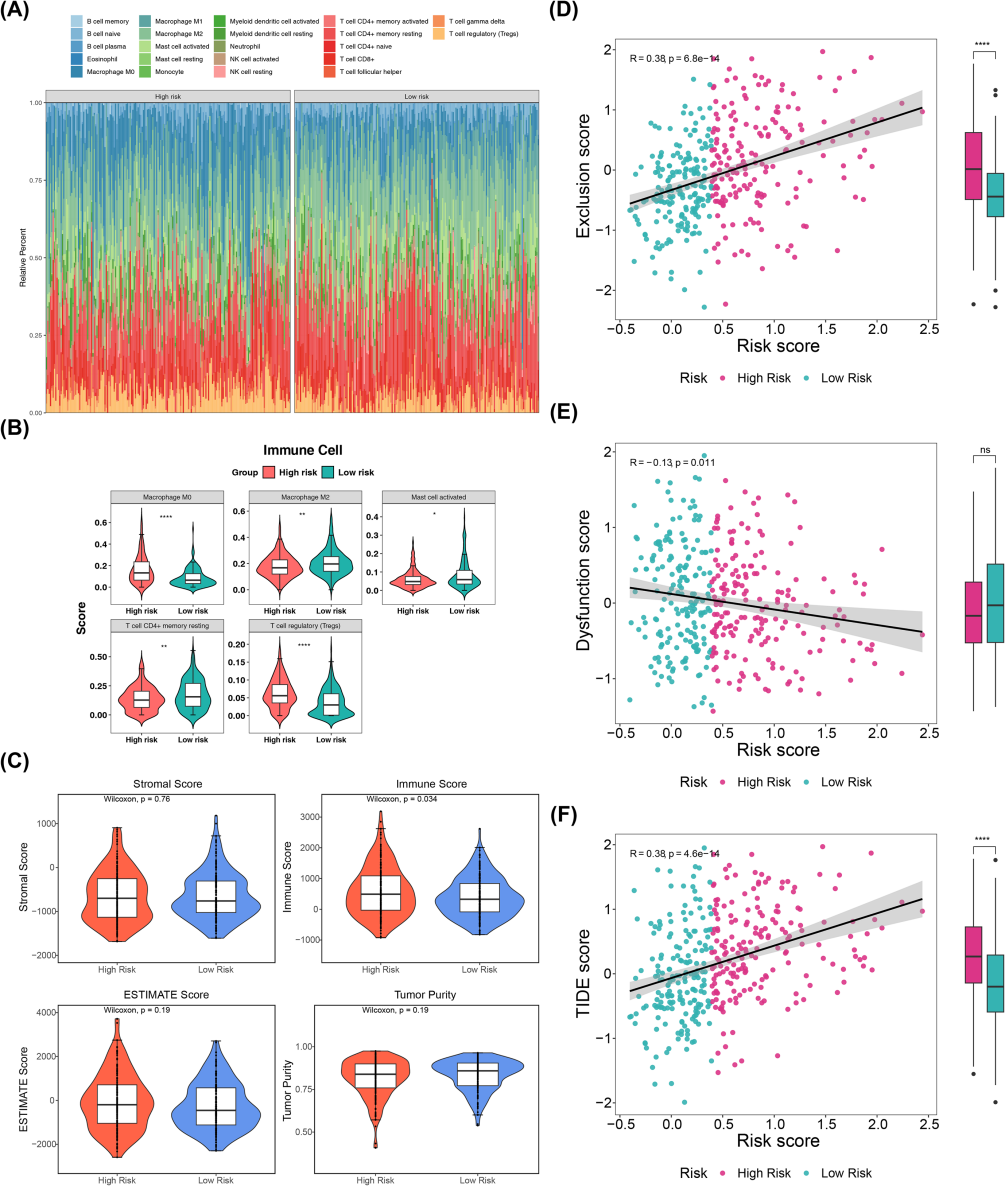
Immune profiling and immune microenvironment dynamics in HCC. **(A)** Heatmap of 22 immune cell populations between risk groups. **(B)** Boxline plots of six differentially infiltrated immune cells. * represents P<0.05, ** represents P<0.01, and **** represents P<0.0001. **(C)** Stromal, ESTIMATE, immune, and tumor scores between risk groups. **(D–F)** Correlation scatter plots, **(D)** between exclusion score and risk score **(E)** between dysfunction score and risk score, **(F)** between TIDE scores and risk score. ns represents P>0.05 and **** represents P<0.0001.

Immune profiling revealed distinct patterns between risk groups, with significant variations in the relative abundance of 22 immune cell populations (**Figure 5A**). Three cell types—activated mast cells, resting memory CD4+ T cells, and M2 macrophages—showed increased infiltration in low-risk patients, while M0 macrophages and Tregs were enriched in high-risk patients (P.adjust< 0.05) (**Figure 5B**). These findings highlight immune microenvironment remodeling between risk groups, suggesting that prognostic genes may serve as potential therapeutic targets for personalized HCC treatment.

ESTIMATE analysis revealed significant inter-group differences in immune scores (**Figure 5C**). High-risk patients exhibited elevated immune exclusion scores, positively correlated with risk scores (**Figure 5D**), while immune dysfunction scores showed a weak negative correlation with risk scores, despite non-significant group differences (**Figure 5E**). TIDE scores were significantly higher in high-risk patients, indicating enhanced immune escape potential (**Figure 5F**). These results suggest that prognostic genes modulate immune microenvironment dynamics and evasion mechanisms, offering novel insights for identifying immunotherapy targets and developing personalized treatment strategies for HCC.

Thirty-nine immune checkpoint genes, including BTLA and BTNL2, displayed significant differential expression between risk groups (**Supplementary Figure 5A**; **Supplementary Table S8**), suggesting that prognostic genes may modulate immune evasion and therapeutic responses through checkpoint regulation. TMB analysis revealed distinct mutation patterns between groups (**Supplementary Figure 5B**). TP53 mutations were observed in 47% of high-risk patients, while CTNNB1 mutations occurred in 34% of low-risk patients. Mutations in genes such as TIN, ALB, MUC16, PCLO, ABCA13, and APOB were present in both groups. Forty TMB genes, including TP53 and SPEG, exhibited higher expression in high-risk patients, while 14 genes, including CTNNB1 and NLRP12, were elevated in low-risk patients (**Supplementary Figure 5C**). Despite similar overall TMB levels between groups (**Supplementary Figure 5D**), prognostic genes may influence mutation profiles through selective regulation of key genes like TP53, providing insights for personalized treatment strategies. Drug sensitivity analysis identified 123 compounds with significantly different IC_50_ values between the risk groups. Seventy drugs (e.g., A.770041, AG.014699) demonstrated enhanced efficacy in high-risk patients (**Supplementary Figure 6A**; **Supplementary Table S9**), while 53 drugs (e.g., A.443654, ABT.263) were more effective in low-risk patients (**Supplementary Figure 6B**). These differential sensitivities likely reflect distinct drug metabolism pathways influenced by prognostic genes, suggesting opportunities for risk-stratified therapeutic optimization in HCC.

### 3.7 Pivotal functional pathways and multiple intercellular interactions of 8 candidate key cells

To explore the related mechanisms at the single-cell level, scRNA-seq data were filtered (**Supplementary Figure 7A**), resulting in an integrated dataset containing 24,863 genes. The degree of variation in these genes is illustrated in **Supplementary Figure 7B**, with the names of the 10 genes exhibiting the highest variation marked. Based on the inflection point plot and PCA replacement test, the top 30 principal components were retained for subsequent analyses (**Supplementary Figure 7C, D**).

**Figure 6:**
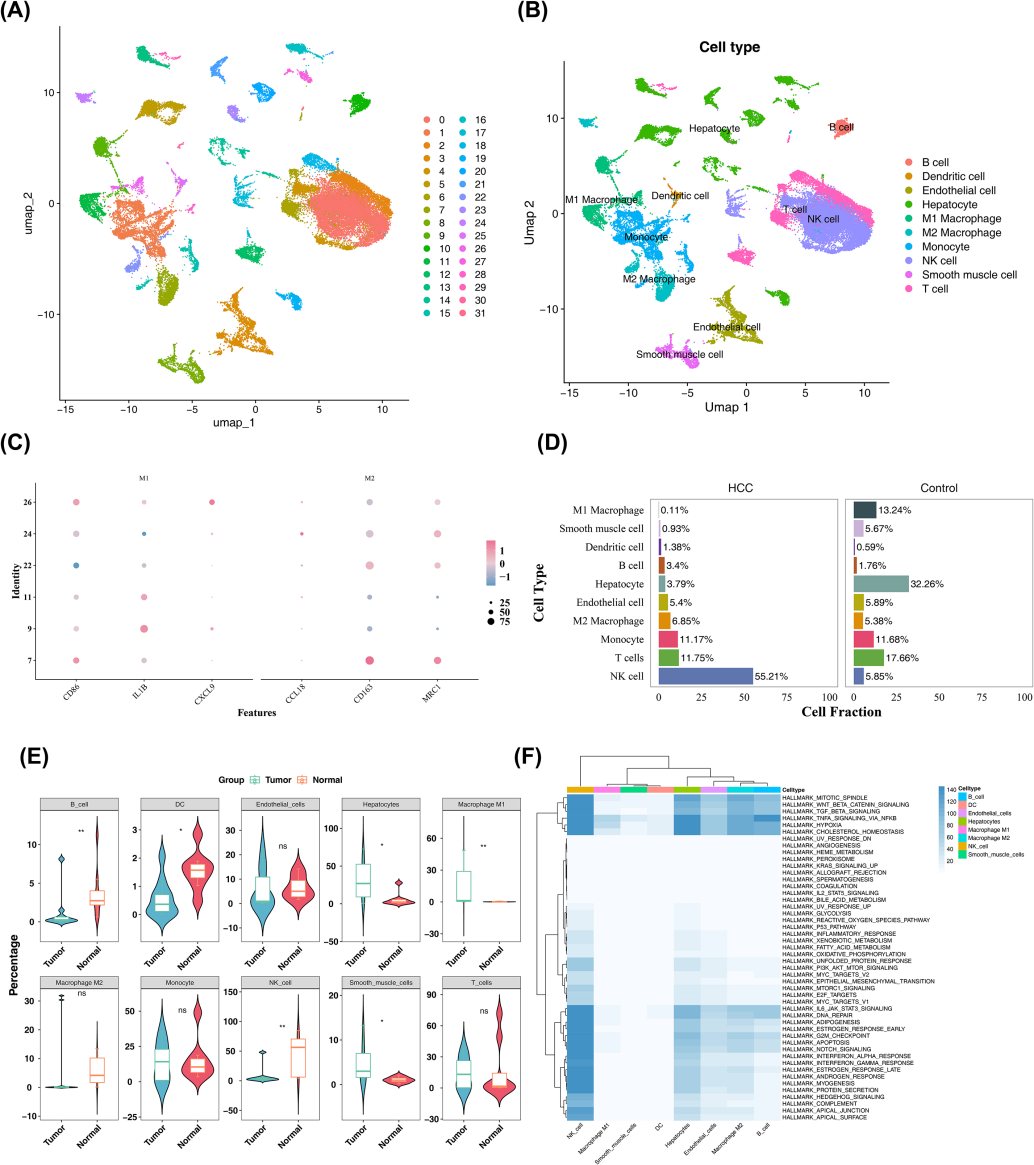
Single-cell transcriptome analysis. **(A, B)** UMAP plots of subclusters **(A)** and annotated cells **(B)**. Different colors distinguish different types of cells. **(C)** Expression of marker genes. The horizontal axis represents the marker gene, the vertical axis shows the recognized cell classification population, and the right side represents the expression level. **(D)** Percent distribution of cell types in HCC and control groups. **(E)** Significant differences (P< 0.05) in 6 cell types between HCC and control groups. ns represents P>0.05, * represents P<0.05, and ** represents P<0.01 **(F)** Pathway enrichment analysis of 8 candidate key cell types.

The retained principal components were clustered and annotated into 32 subclusters. These cells were classified into 10 types, including dendritic cells, B cells, endothelial cells, hepatocytes, M1 macrophages, M2 macrophages, monocytes, NK cells, smooth muscle cells, and T cells (**Figures 6A–C**). Notably, 25 M1 macrophages and 1,533 M2 macrophages were identified. The percentage of each cell type in HCC and control groups is shown in **Figure 6D**, with significant differences observed between the groups in six cell types, including dendritic cells, B cells, hepatocytes, M1 macrophages, NK cells, and smooth muscle cells (P< 0.05) (**Figure 6E**). Considering that statistical analyses were performed on individual samples, and the differential trends of M2 macrophages and endothelial cells and their critical roles in HCC progression, these cell types, along with the six previously mentioned cell types, were selected as candidate key cells for further analyses. Subsequently, the eight candidate key cell types were enriched in pathways such as TNFA signaling *via* NFKB and hypoxia, which are closely linked to HCC progression (**Figure 6F**). This suggests that these candidate key cells may significantly influence HCC progression through these pathways.

Finally, the interactions between the eight candidate key cell types and other cell types were explored (**Supplementary Figures 8A–C**). The results revealed numerous and high-intensity interactions involving hepatocytes, endothelial cells, M1 macrophages, and M2 macrophages in both HCC and control groups, suggesting that these cells play central roles in the cell-cell communication networks of the HCC microenvironment. Furthermore, the interaction between M2 macrophages and smooth muscle cells was more prominent in both groups, indicating that this interaction is especially crucial within the communication network. These findings highlight that hepatocytes and M2 macrophages are core components mediating cellular crosstalk in the HCC microenvironment, providing a structural basis for the metabolic-immune interplay between these two cell types.

### 3.8 Trajectories of hepatocyte and M2 macrophage and the expression patterns of prognostic genes

First, the expression of prognostic genes across different annotated cell types in the scRNA-seq dataset was analyzed (**Supplementary Figure 9A**). Eight prognostic genes—PFKFB4, ADH4, ADH1C, ME1, FOXK1, PFKP, ARL2, and TKTL1—were significantly differentially expressed in hepatocytes between HCC (tumor) and control (normal) groups, while four prognostic genes—ADH1C, ME1, PFKP, and TKTL1—were significantly differentially expressed in M2 macrophages between the groups (**Supplementary Figure 9B**). Based on these findings, hepatocytes and M2 macrophages were selected as key cells for further analysis. Changes in gene expression in hepatocytes could reflect the characteristics of HCC cells, while alterations in M2 macrophages may reveal changes in the HCC microenvironment.

**Figure 7:**
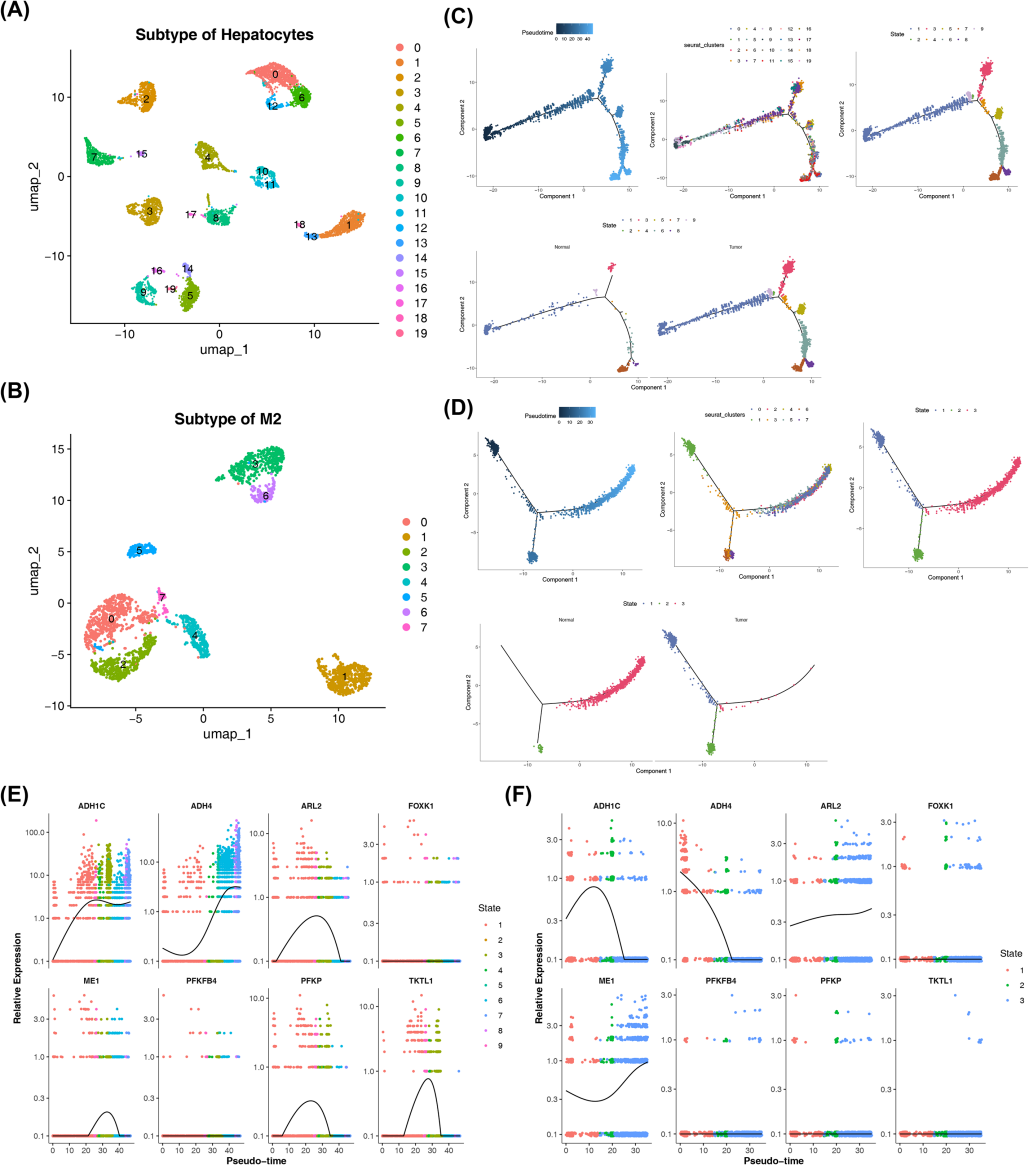
Subpopulation clustering and trajectory analysis of hepatocytes and M2 macrophages. **(A, B)** Clustering of hepatocytes and M2 macrophages. **(C-D)** Cell trajectory analysis of hepatocytes and M2 macrophages. **(E, F)** Prognostic gene expression patterns at various differentiation states in hepatocytes and M2 macrophages.

Next, hepatocytes and M2 macrophages were clustered into 20 and 8 subpopulations, respectively, indicating significant heterogeneity within these two key cell types in both HCC and control groups (**Figures 7A, B**). Subsequently, cell trajectory analysis was performed on the subpopulations of these two cell types. The trajectories of hepatocytes and M2 macrophages were inferred (**Figures 7C, D**). Hepatocytes and M2 macrophages exhibited 9 and 3 distinct differentiation states, respectively. Notably, hepatocytes in the HCC group were more differentiated, while M2 macrophages in the control group showed more differentiation, suggesting that hepatocytes in HCC are more developed, which may reflect their complex role in HCC progression. Additionally, M2 macrophages in the HCC group were predominantly in the early stages of differentiation, whereas those in the control group were mainly in the later stages, implying that M2 macrophages in HCC exhibit early differentiation features that may influence the TME.

Furthermore, the expression of prognostic genes in hepatocytes and M2 macrophages was examined at different states of differentiation (**Figures 7E, F**). In hepatocytes, the expression of ARL2, PFKP, ME1, and TKTL1 showed an initial increase followed by a decrease across the differentiation states, while ADH1C exhibited a continuous increase. The expression of ADH4 demonstrated a decrease followed by an increase. However, no significant sequential changes were observed in the expression of FOXK1 and PFKFB4. In M2 macrophages, the expression of ADH1C showed a trend of increase followed by a decrease, while ADH4 expression showed a continuous decrease. ME1 expression decreased initially before increasing. In contrast, the expression of FOXK1, PFKFB4, PFKP, and TKTL1 did not display any sequential changes across the differentiation states in M2 macrophages. These differences in differentiation states and the dynamic changes in gene expression provide insights into the potential interconnected functional remodeling processes that hepatocytes and M2 macrophages undergo during the progression of HCC.

## 4 Discussion

HCC is a prevalent and aggressive malignancy that often leads to a poor prognosis due to its invasive characteristics. Glycolysis and M2 macrophages are strongly linked to HCC progression. This study integrated bulk transcriptomic data with scRNA-seq to systematically explore the prognostic significance of the glycolysis–M2 macrophage interaction axis in HCC. While previous research has independently highlighted the roles of metabolic reprogramming and tumor-associated macrophages in HCC, the dynamic interplay between glycolysis and M2 macrophages and their combined prognostic value have not been fully characterized.

This study identified eight prognostic genes associated with both glycolysis and M2 macrophages (PFKFB4, ADH4, ADH1C, ME1, FOXK1, PFKP, ARL2, and TKTL1) and developed a risk score model based on this metabolic–immune interaction axis. This model demonstrated strong prognostic performance, which was further validated in an independent cohort. Additionally, through single-cell resolution analyses, the expression dynamics and cell state-dependent patterns of these genes in hepatocytes and M2 macrophage subpopulations were characterized. These results suggest that metabolic reprogramming, particularly through key intermediates such as lactate, may influence M2 macrophage polarization, thereby reshaping the tumor immune microenvironment.

Overall, this integrative analytical approach not only enhances the understanding of immunometabolic crosstalk in HCC but also moves beyond conventional prognostic models that focus solely on metabolic or immune features, offering valuable mechanistic insights.

### 4.1 Category 1: glycolysis-related genes (PFKFB4, PFKP, ME1, TKTL1)

These four metabolic enzyme genes play pivotal roles in tumor metabolic reprogramming. PFKFB4 regulates glycolysis and pentose phosphate production. As a downstream target of THOC3, PFKFB4 mediates tumorigenic effects in lung squamous cell carcinoma (LUSC) cells. Knockout of PFKFB4 inhibits cell proliferation, migration, and significantly reduces glucose uptake, lactate production, and intracellular ATP levels (35). PFKP, an isoenzyme of phosphofructokinase-1 (PFK-1), regulates cell proliferation, apoptosis, autophagy, migration, and stemness. Silencing PFKP effectively reduces HCC cell proliferation and decreases β-catenin levels (20, 36, 37). ME1, a cytoplasmic enzyme that converts malate into pyruvate, generates NADPH and promotes the Warburg effect in cancer cells. Under hypoxic conditions, overexpression of ME1 enhances epithelial-mesenchymal transition (EMT) and promotes tumor budding (38, 39). TKTL1, the bottleneck enzyme in the non-oxidative pentose phosphate pathway, fuels ESCC growth and spread. Knockout of TKTL1 effectively inhibits cell proliferation and promotes apoptosis (40).

In the present study, several GRGs, including PFKFB4 and TKTL1, were highly expressed in high-risk patients with HCC, reflecting enhanced glycolytic flux and increased lactate production in tumor cells. Emerging evidence indicates that lactate, rather than being just a metabolic byproduct, serves as a key signaling molecule in the TME (41). Lactate has been shown to induce histone H3K18 lactylation, directly activating the transcription of M2 macrophage–associated genes such as CD206 and ARG1, thereby promoting macrophage polarization toward the tumor-promoting M2 phenotype (42).

Our data indicated that M2 macrophage infiltration was higher in the low-risk group, suggesting a relatively restrained glycolytic activity and a less lactate-enriched microenvironment, which may limit M2 polarization. In contrast, the elevated expression of GRGs in the high-risk group fosters a lactate-rich TME, facilitating M2 macrophage polarization and establishing an immunosuppressive niche that promotes HCC progression. These findings provide mechanistic insights into the role of the “glycolysis–lactate–M2 macrophage” axis in shaping the tumor immune microenvironment and influencing HCC prognosis.

### 4.2 Category 2: alcohol dehydrogenase family genes (ADH4 and ADH1C)

ADH4 and ADH1C, key members of the alcohol dehydrogenase (ADH) family, metabolize a variety of substrates, including retinol and ethanol. ADH4 is suggested to interact with immune cells, influencing HCC progression, prognosis, and treatment response, making it a critical biomarker for early disease detection and prognostic assessment (43). ADH1C, also a member of the ADH family, regulates the PHGDH and PSAT1/serine metabolism pathway, inhibiting colorectal cancer (CRC) development. Overexpression of ADH1C suppresses CRC cell growth, motility, tissue invasion, and tumor colony formation (44).

In HCC, multiple database analyses and immunohistochemical experiments reveal that ADH4 is significantly downregulated in tumor samples, with low expression correlating strongly with poor outcomes. Patients with low ADH4 expression have shorter overall survival, relapse-free survival, cancer-specific survival, and progression-free survival (43). Mechanistic studies indicate that, in normal liver tissue, MEG3 normally sequesters miR-664a-3p, preventing its suppression of ADH4. However, in HCC, downregulation of MEG3 leads to upregulation of miR-664a-3p, which suppresses ADH4 expression (45). ADH1C, along with CXCL11, EMCN, SPARCL1, and LIN28B, forms a TACE failure signature (TFS) that can predict the response of patients with HCC to TACE treatment. As a core gene of the TFS, low expression of ADH1C regulates the immune microenvironment and metabolic pathways involved in HCC resistance to TACE treatment (46). RT-qPCR validation confirmed that both ADH4 and ADH1C were significantly downregulated in HCC tissues, aligning with the trends observed in dataset analyses and further validating the accuracy of these findings.

### 4.3 Category 3: transcriptional regulation and cellular function genes (FOXK1 and ARL2)

FOXK1, a member of the evolutionarily conserved forkhead box transcription factor family, is upregulated in HCC cells compared to normal liver cells, with its downregulation reducing cell viability (47). Further studies have shown a marked increase in both mRNA and protein levels of FOXK1 in human HCC tissues, with its expression higher in HCC cells than in normal liver cells. In a nude mouse xenograft model, tumors formed by FOXK1-knockdown HepG2 cells were significantly smaller in both volume and weight compared to the control group, suggesting FOXK1 promotes HCC growth *in vivo* (48). Hepatocyte-specific knockout of FOXK1 in the non-alcoholic steatohepatitis (NASH) model resulted in a significant reduction in liver tumor size and number, as well as prolonged survival, indicating that the loss of FOXK1 inhibits HCC development (49).

ARL2, a small GTPase from the Ras superfamily, is widely expressed in various tissues. Studies suggest that miR-497 may target ARL2 in hepatocytes, with higher ARL2 levels associated with poorer outcomes in patients with HCC (50). Further mechanistic studies revealed that ARL2 interacts directly with GAS6-AS2 and miR-3619-5p, and rescue experiments showed that overexpressing ARL2 can reverse the suppression of HCC cells induced by miR-3619-5p or GAS6-AS2 deletion (51). Additionally, tumors formed by ANRIL-overexpressing Huh7 cells in nude mice exhibited increased ARL2 expression, upregulation of Cyclin A1 and Cyclin B1, and accelerated tumor growth (52). In the present study, PCR validation showed a significant upregulation of FOXK1 expression in HCC tissues, while ARL2 did not show significant differences, likely due to the small sample size. Nonetheless, these findings suggest potential directions for future research.

The prognostic risk model constructed from the eight identified genes demonstrated stable and reproducible predictive performance in both the TCGA-LIHC training cohort and the ICGC validation cohort. Time-dependent ROC analyses revealed AUC values exceeding 0.60 at 1, 3, and 5 years in both datasets, with an average AUC of 0.72 in the training cohort and 0.68 in the validation cohort, indicating strong discriminative ability and cross-cohort generalizability. To further assess the predictive performance of our model, it was compared with previously reported HCC prognostic signatures. For instance, the six-gene risk model proposed by Gao et al. achieved AUC values of 0.73, 0.63, and 0.64 at 1, 3, and 5 years, respectively (average AUC = 0.67) (53), while the seven-gene model by Yang et al. showed AUC values of 0.74, 0.65, and 0.63 at 1, 2, and 3 years (average AUC = 0.67) (54). Compared to these models, our risk score demonstrated comparable or superior predictive performance across multiple time points. Importantly, our model is uniquely grounded in the biological interplay between glycolysis and M2 macrophages, providing additional mechanistic insights beyond statistical prognostic signatures. However, the current model was primarily derived and validated using retrospective public datasets. Prospective validation in large, multicenter clinical cohorts is essential to further confirm its robustness and clinical applicability in real-world settings.

Conventional serum biomarkers, such as AFP, are commonly used for auxiliary diagnosis and disease monitoring in HCC, but their prognostic value remains limited, particularly in predicting responses to immunotherapy. Emerging liquid biopsy biomarkers, including circulating tumor DNA and exosomal RNA, hold promise; however, they still face challenges related to technical standardization and clinical validation (55). In this context, the eight-gene signature identified in our study (which includes PFKFB4 and ADH4) offers a functional perspective, capturing two core biological processes underlying HCC progression: metabolic reprogramming driven by enhanced glycolysis and immune microenvironment remodeling marked by M2 macrophage polarization. This model not only provides independent prognostic information but also reflects potential mechanisms of therapeutic resistance, such as glycolysis-driven metabolic adaptation and immune evasion through immunosuppressive macrophage phenotypes. Beyond prognostic stratification, this functional integration could offer biologically informed guidance for therapeutic decision-making.

As shown in **Figure 1B**, the relative infiltration of M2 macrophages was lower in HCC tissues compared to adjacent non-tumor tissues, while M0 macrophages and Tregs were increased. This observation highlights the complexity of the HCC immune microenvironment. The increase in M0 macrophage infiltration may reflect continuous immune cell recruitment, while changes in M2 macrophage proportions may indicate functional reprogramming or altered cellular interactions within the TME (56, 57). Notably, our focus on M2 macrophages was not solely based on their infiltration levels but rather on their functional coupling with tumor glycolytic metabolism. Even when present at relatively low overall proportions, metabolically active M2 macrophage subpopulations localized within specific tumor niches can exert disproportionate pro-tumorigenic effects through close interaction with highly glycolytic cancer cells (58). By integrating M2 polarization-related gene sets with glycolysis-associated genes, and further validating their co-expression and interaction networks at the single-cell level, our findings support the functional relevance and prognostic significance of a “hepatocyte–glycolysis–M2 macrophage” axis in HCC. Additionally, the prominent infiltration of M0 macrophages and Tregs suggests that these immune populations also play critical roles in the metabolism–immunity interplay in HCC and warrant independent investigation in future studies.

Furthermore, although the TIDE and ESTIMATE algorithms were employed to assess the immune microenvironment in HCC, their potential limitations must be acknowledged. TIDE infers immune evasion potential based on bulk transcriptomic data and may not fully capture HCC-specific immunoregulatory features, such as the influence of hepatocyte-derived metabolic products (e.g., lactate) on immune cell function (59). Additionally, TIDE primarily relies on the expression of known immune checkpoint-related genes and may not encompass emerging immunosuppressive pathways specific to HCC. ESTIMATE estimates immune and stromal scores, as well as tumor purity; however, in HCC tissues, tumor purity can be substantially affected by hepatocyte-rich parenchyma, fibrosis, and necrotic regions, potentially introducing bias into microenvironmental assessments (59, 60). Future studies integrating multi-region sampling, histopathological evaluation, or spatial transcriptomics may help correct for tumor purity effects and further enhance the interpretability and applicability of immunogenomic models in HCC.

Cell–cell communication analysis revealed that hepatocytes and M2-polarized macrophages serve as central interaction hubs within the HCC TME, establishing a structural basis for metabolic–immune crosstalk. Integrating these findings with existing literature, this study proposes that hepatocytes with high expression of GRGs, such as PFKFB4 and TKTL1, may generate excessive metabolic intermediates, particularly lactate, which act as signaling molecules influencing neighboring immune cells. Lactate has been shown to promote the transcription of M2 macrophage–associated genes through epigenetic mechanisms, such as histone H3K18 lactylation, driving macrophage polarization toward an immunosuppressive phenotype (42).

Trajectory analysis provided further dynamic insights into this interaction. Hepatocytes in HCC exhibited significant differentiation heterogeneity, with stage-dependent changes in GRG expression, indicating that metabolic reprogramming evolves alongside tumor progression. This temporal and spatial metabolic heterogeneity may, in turn, reshape the immune microenvironment by modulating macrophage functional states through sustained cell–cell communication. Notably, our single-cell analyses demonstrated that the identified prognostic genes exhibited preferential expression in hepatocytes and M2 macrophage subsets, rather than being uniformly expressed across all cell types. This supports their cell-type–specific relevance in HCC.

These single-cell findings extend beyond descriptive observations, proposing a mechanistic framework where aberrantly activated tumor glycolysis alters the local metabolic milieu. Through intensive intercellular communication, this process influences M2 macrophage differentiation and function. This coordinated metabolic–immune interaction may contribute to immune evasion and tumor progression in HCC and provides a theoretical rationale for future therapeutic strategies targeting the “glycolysis–M2 macrophage axis,” such as combining metabolic inhibitors with immune checkpoint blockade.

The prognostic genes identified in this study may also guide combinatorial strategies for targeted therapy and immunotherapy in HCC. Several key glycolytic enzymes, including PFKFB4 and ME1, have been identified as druggable targets, with small-molecule inhibitors demonstrating antitumor activity in preclinical models (38, 61, 62). Inhibition of these GRGs may not only suppress tumor cell proliferation but also reduce lactate production, thereby alleviating M2 macrophage–driven immunosuppression within the TME.

Furthermore, M2-polarized macrophages themselves represent important therapeutic targets. Strategies such as CSF1R inhibition or CD47 blockade have been shown to deplete or reprogram tumor-associated macrophages toward antitumor phenotypes. Notably, our findings indicate that low expression of ADH4 is associated with poor prognosis in HCC. ADH4 has been linked to retinol metabolism and immune regulation, suggesting that its dysregulation may contribute to immunosuppressive signaling pathways (43). These findings provide a theoretical rationale for exploring metabolic interventions in combination with immune checkpoint inhibitors, such as anti–PD-1 therapy.

In conclusion, our results support the concept that targeting tumor glycolysis in conjunction with macrophage-directed therapies or immune checkpoint blockade could offer a promising combinatorial strategy for high-risk patients with HCC. Future experimental and clinical studies are required to validate the efficacy and safety of such “glycolysis inhibition plus immunotherapy” approaches.

The primary innovation of this study lies in systematically elucidating the integrated prognostic value of the glycolysis–M2 macrophage interaction axis in HCC. Through comprehensive bioinformatics analyses, a set of prognostic genes jointly linked to metabolic reprogramming and the immune microenvironment was identified, and a stable and reliable risk model with predictive capacity was constructed. Furthermore, by incorporating single-cell transcriptomic data, the dynamic expression patterns of these genes in hepatocytes and M2-polarized macrophages were characterized, providing mechanistic insights into metabolic–immune crosstalk during HCC progression. Collectively, these findings extend beyond single-dimensional analyses of metabolism or immunity, offering a novel framework for prognostic assessment and a theoretical basis for therapeutic strategies targeting the immunometabolic axis in HCC.

However, this study has several limitations. First, the identified prognostic genes have not been functionally validated through experimental assays, such as proliferation, migration, and invasion analyses, to directly confirm their biological roles in HCC. Second, the conclusions drawn are primarily based on retrospective analyses of publicly available datasets, and the lack of validation in independent prospective clinical cohorts may limit the generalizability of the findings. Third, the inference of M2 macrophage–related features relied on computational deconvolution approaches, such as CIBERSORT, which may be influenced by tumor heterogeneity, stromal contamination, and variations in tumor purity. Finally, the predicted drugs still need to be further explored for their efficacy in HCC through *in vitro* experiments.

To address these limitations, future studies are necessary. First, functional gain- and loss-of-function experiments using HCC cell lines and animal models should be conducted to elucidate the direct roles of the identified prognostic genes in regulating malignant phenotypes and the metabolic–immune microenvironment. Second, multicenter, prospective HCC cohorts with comprehensive clinical follow-up should be collected to independently validate the prognostic risk model and further evaluate its translational potential. Third, integrative experimental approaches, including multiplex immunofluorescence and flow cytometry, should be employed to directly quantify and spatially localize M2 macrophages within tumor tissues, complementing and validating bioinformatics-based inferences while enabling a more precise characterization of their pathological interactions with tumor glycolysis. In addition, *in vitro* experiments will be carried out in HCC cell lines to detect the effects of the predicted drugs on cell proliferation, apoptosis and invasion, and to validate the interactions of the drugs with the eight prognostic genes, so as to provide experimental basis for the clinical precision of drug administration.

## Data Availability

The original contributions presented in the study are included in the article/**Supplementary Material**. Further inquiries can be directed to the corresponding author.
