# Supplementary material for: Identification and validation of prognostic genes related to glycolysis and M2 macrophage in hepatocellular carcinoma: an integrated analysis of bulk RNA sequencing and single-cell RNA sequencing
Source: Front Immunol. 2026 Feb 12;17:1710411. doi: 10.3389/fimmu.2026.1710411 (PMC12935947; doi:10.3389/fimmu.2026.1710411)
Supplement: Supplementary file 1 [file Table1.docx]

Supplementary Material

# Supplementary Material

## Supplementary Figures


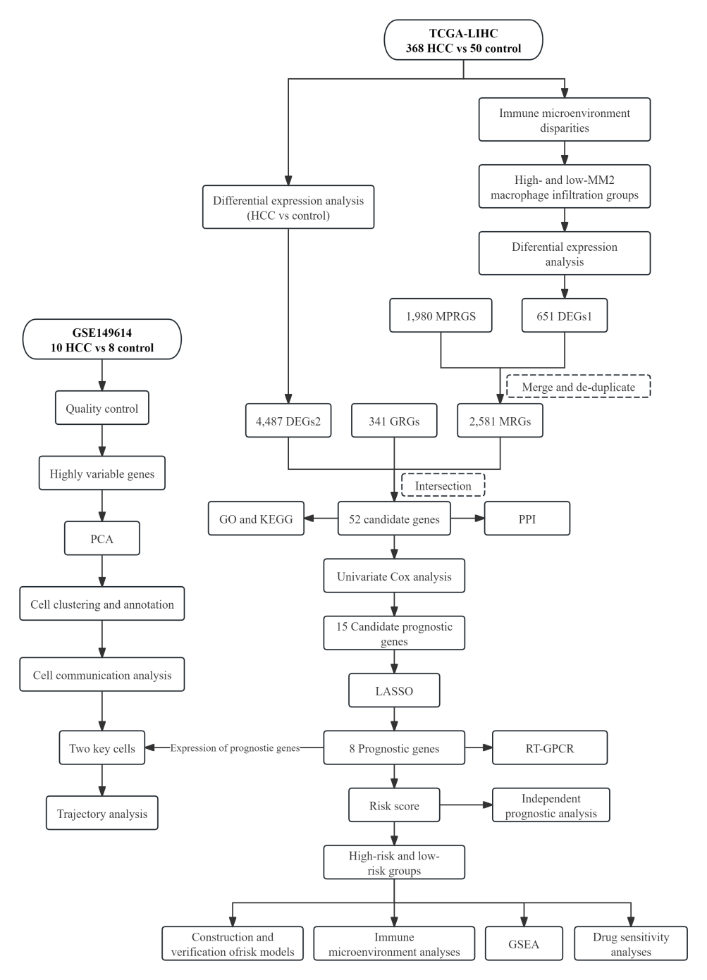


**Supplementary Figure 1.** Analyze the flowchart.

**
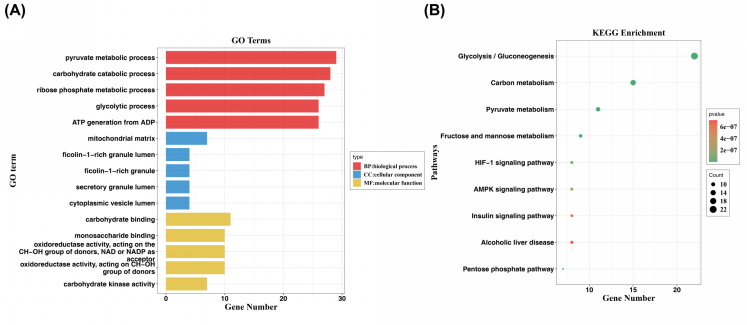
**

**Supplementary Figure 2.** GO and KEGG enrichment analysis of candidate genes. (**A**) Enrichment of candidate genes in GO terms and (**B**) KEGG pathways.


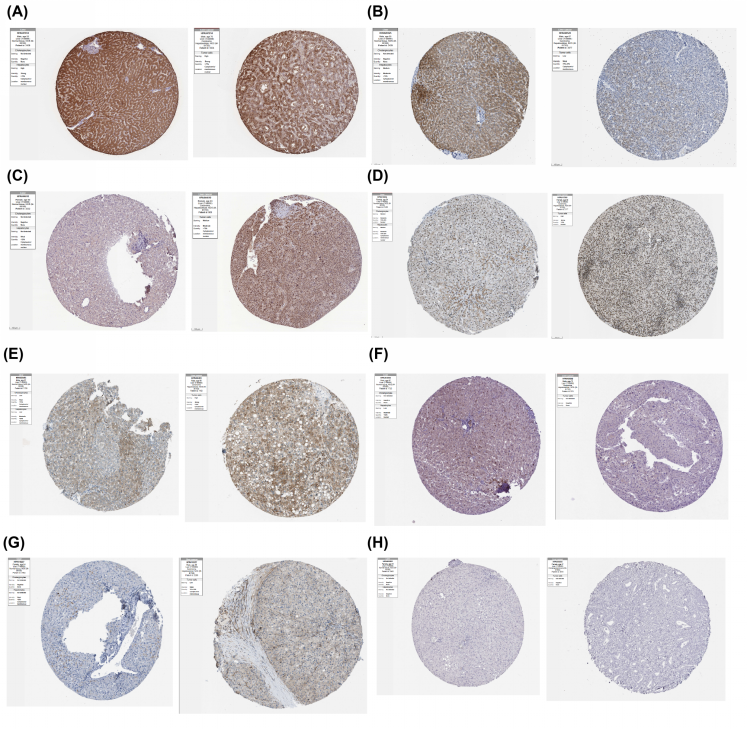


**Supplementary Figure 3**. Analysis of protein expression levels of prognostic genes. (A) ADH1C. (B) ADH4. (C) ARL2, (D) FOXK1. (E) ME1. (F) PFKFB4. (G) PFKP. (H) TKTL1.


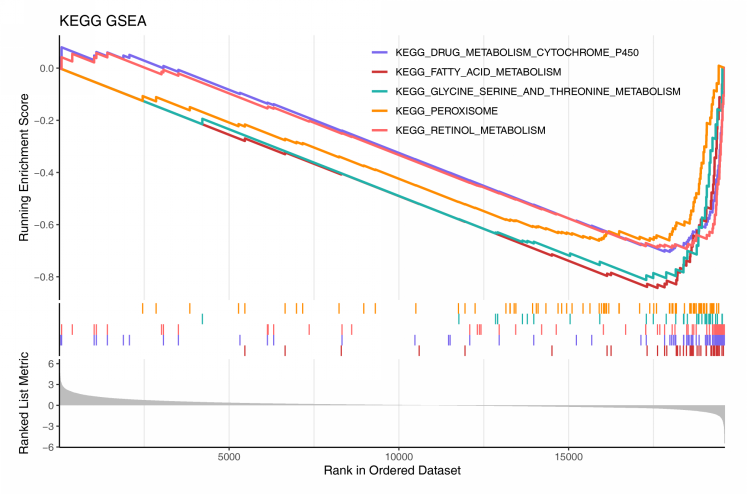


**Supplementary Figure 4.** GSEA results of two risk groups.


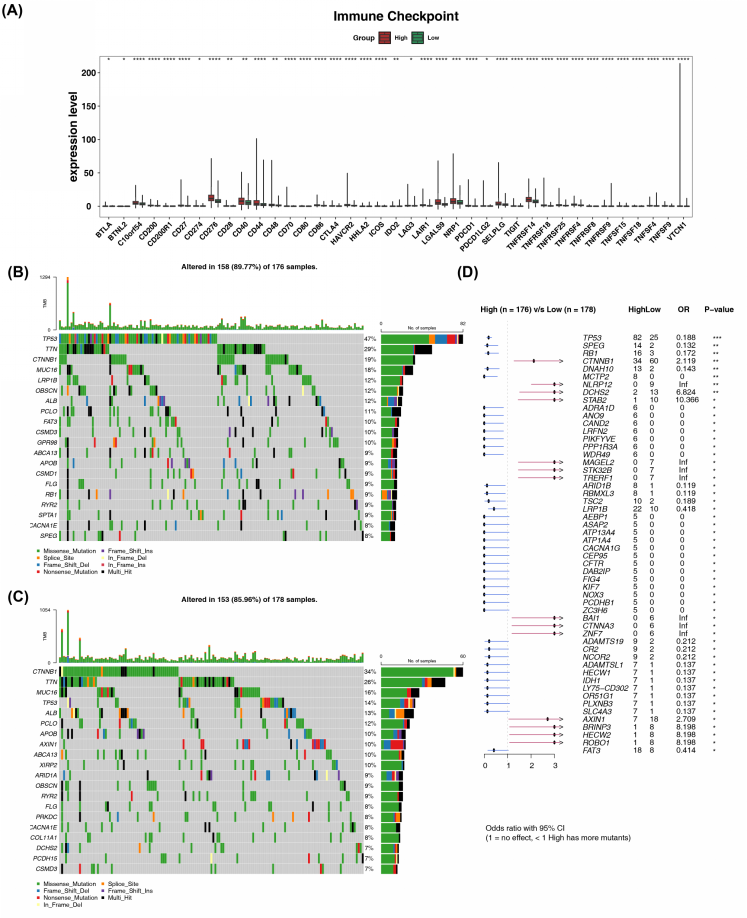


**Supplementary Figure 5**. Immune checkpoint expression and tumor mutational landscape in HCC risk groups. (**A**) Differential expression of 39 immune checkpoint genes between high- and low-risk patients. (**B-C**) Mutational analysis waterfall diagram for high-risk (**B**) and low-risk (**C**) groups. (**D**) Genes with significant mutations between high-risk and low-risk groups. * represents P < 0.05, ** represents P < 0.01, and *** represents P < 0.001.


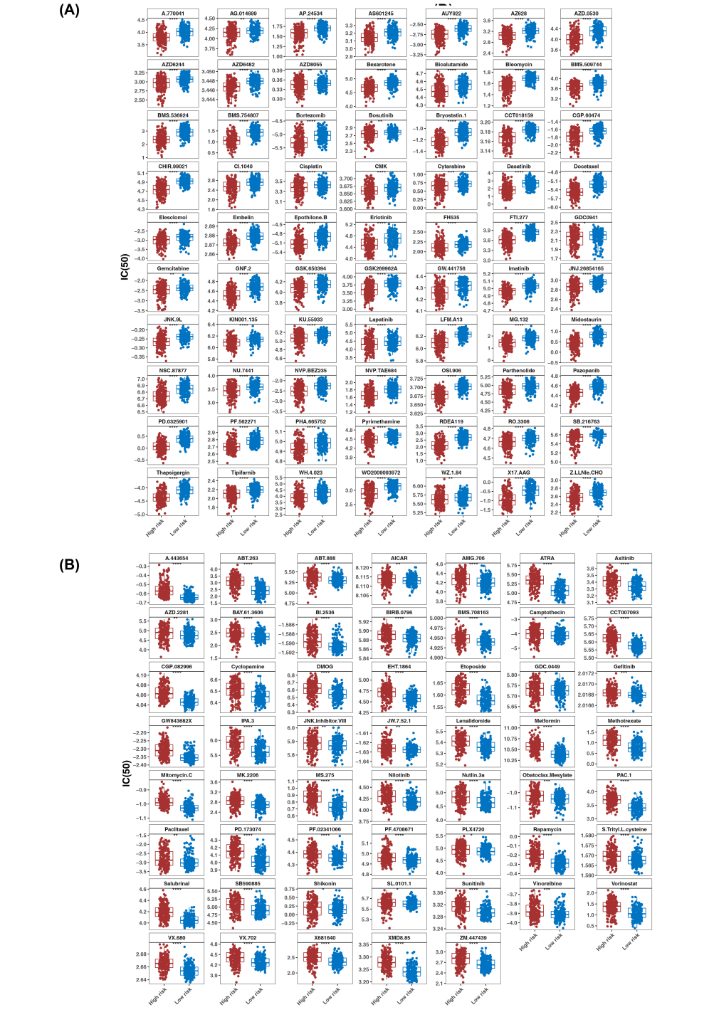


**Supplementary Figure 6**. Drug sensitivity profiles across HCC risk groups. (**A**) High-risk patients. (**B**) Low-risk patients. * indicates P<0.05, ** indicates P<0.01, *** indicates P<0.001, and **** indicates P<0.0001


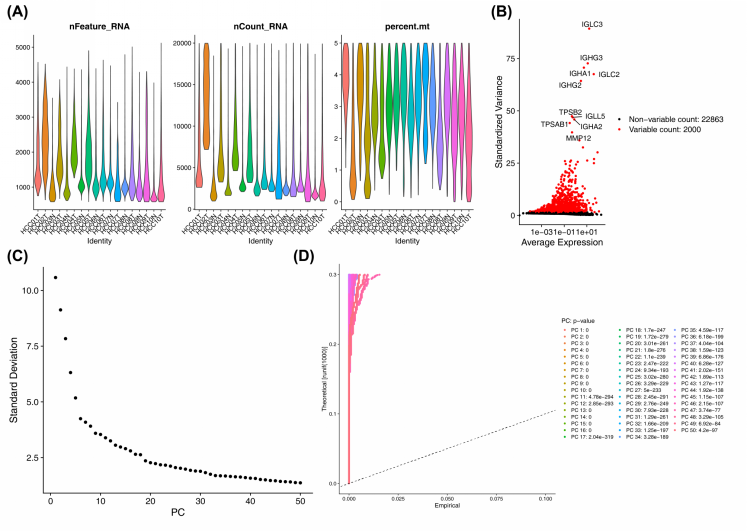


**Supplementary Figure 7**. Data processing for single-cell analysis. (**A**) Filtering of scRNA-seq data for further analysis. (**B**) Selected for high variable genes. (**C-D**) Retention of the top 30 principal components (PCs) for subsequent analyses.


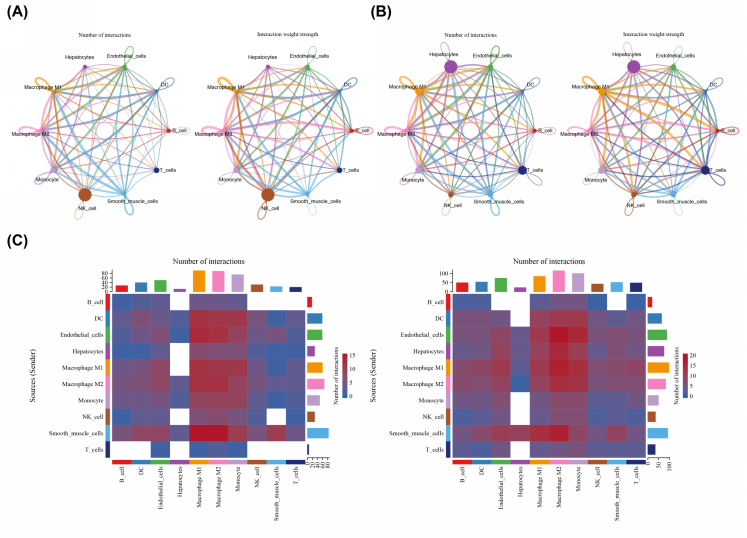


**Supplementary Figure 8**. Cell-cell communication analysis. **(A-B)** Cell-cell interaction networks in HCC and control groups. The arrows indicate the direction, and the thickness of the lines represents the quantity (left) or weighting (right) of interactions between cells. **(C)** Heatmaps of cell communication in HCC and control groups.


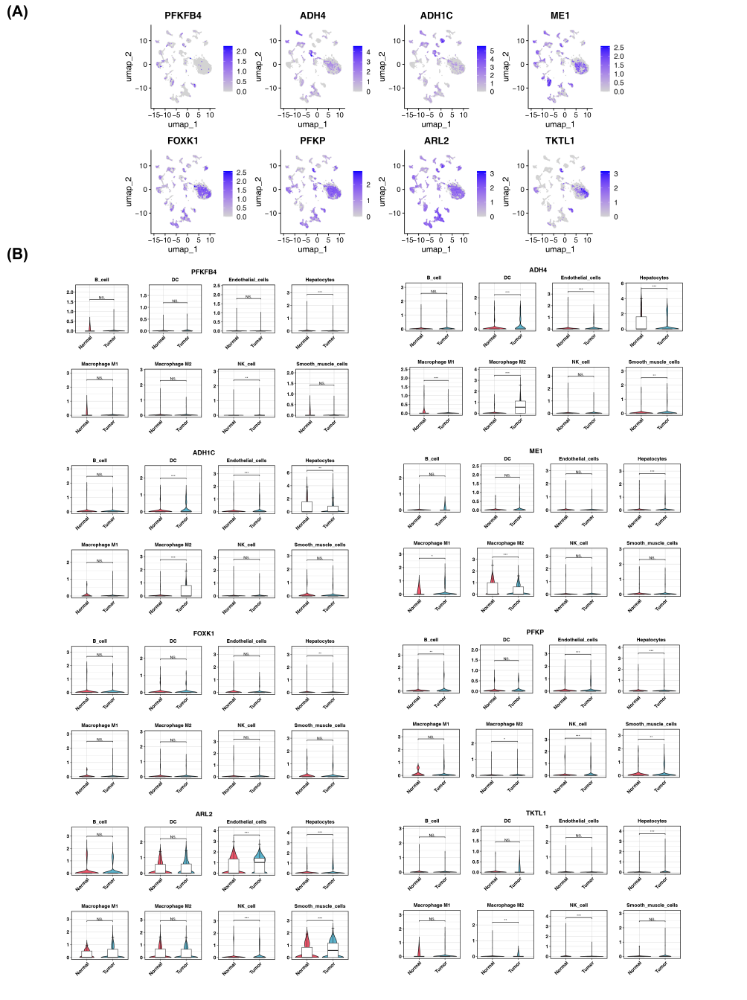


**Supplementary Figure 9**. Differential Expression of Prognostic Genes in Key Cell Types. **(A)** UMAP plots displayed the expression of 8 prognostic genes in different annotated cell types from the scRNA-seq dataset. **(B)** Significant differential expression of 8 prognostic genes (PFKFB4, ADH4, ADH1C, ME1, FOXK1, PFKP, ARL2, and TKTL1) in different cell types.

## Supplementary Tables

**Supplementary Table 1.** Primer sequence of RT-qPCR.

| **Primers** | **Sequences (5’ to 3’)** | **Bp** |
| --- | --- | --- |
| PFKFB4-F | GTCCCCACGGGAATTGACAC | 135 |
| PFKFB4-R | AGGCCCACCATGACAATGAG |  |
| ADH4-F | GCAAGCCCCTTTGCATTGAA | 171 |
| ADH4-R | CCACAATACCTGCAGCCTCA |  |
| ADH1C-F | CCCAAACTTGTGGCTGACTTT | 117 |
| ADH1C-R | ACTCTTTCCAGAGCGAAGCA |  |
| ME1-F | CACAGCCTGAGCAGTGAGAA | 149 |
| ME1-R | AGGCCATTCTGGCAGGTAAC |  |
| FOXK1-F | CCCGTGTCCCGTTGTTTTTC | 102 |
| FOXK1-R | GCAACAGGTACGGACTTCCA |  |
| PFKP-F | TGCAGCAGAGGATGGGATTG | 263 |
| PFKP-R | CCCTCCAGTCACCCCTTACA |  |
| ARL2-F | GCACTGTCCTCTAACGCCAT | 211 |
| ARL2-R | GTGAAGGTTGAGGGACCTGG |  |
| TKTL1-F | CGTGTGCTAGGAAGCCAGTT | 238 |
| TKTL1-R | TCCACATGCAACTCCCAGTC |  |
| GAPDH-F | ATGGGCAGCCGTTAGGAAAG | 135 |
| GAPDH-R | AGGAAAAGCATCACCCGGAG |  |

| **Component** | **Volume** |
| --- | --- |
| 5x Reaction Buffer | 4 ul |
| Primer | 1 ul |
| SweScript RT I Enzyme Mix | 1 ul |
| Total RNA | 2 ug |
| Nuclease-Free Water | Add to 20 ul |

**Supplementary Table 2.** Reagents used in RT-qPCR.

**Supplementary Table 3.** Reaction temperature in RT-qPCR.

| **Temperature** | **Time** |
| --- | --- |
| 25℃ | 5 min |
| 50℃ | 15 min |
| 85℃ | 5 s |
| 4℃ | hold |

**Supplementary Table 4.** Component in PCR.

| **Component** | **Volume** |
| --- | --- |
| cDNA | 3 ul |
| 2x Universal Blue SYBR Green qPCR Master Mix | 5 ul |
| Forward primer (10 µM) | 1 ul |
| Reverse primer (10 µM) | 1 ul |

**Supplementary Table 5.** PCR process.

| **Process** | **Temperature** | **Time** |
| --- | --- | --- |
| Initial denaturation | 95℃ | 1 min |
| Denaturation | 95℃ | 20 s |
| Annealing | 55℃ | 20 s |
| Extension | 72℃ | 30 s |

**Supplementary Table 6.** The differential expression of eight prognostic genes between HCC samples and control samples.

| GeneSymbol | baseMean | log2FoldChange | lfcSE | stat | pvalue | padj | change |
| --- | --- | --- | --- | --- | --- | --- | --- |
| PFKFB4 | 180.49107894949 | 1.63616452990971 | 0.192679777957158 | 8.49162557304541 | 2.03766596160003e-17 | 2.04199681380322e-16 | UP |
| ADH4 | 43233.6575189173 | -2.358613826 | 0.315144347220529 | -7.484233328 | 7.19660009141166e-14 | 5.06069641029172e-13 | DOWN |
| ADH1C | 27658.0783497828 | -1.527399238 | 0.294077460322753 | -5.193867074 | 2.05970229058589e-07 | 6.76877246914964e-07 | DOWN |
| ME1 | 1201.38208705784 | 1.0119828773815 | 0.232213198073827 | 4.35799035444905 | 1.31262167305198e-05 | 3.42170625483903e-05 | UP |
| FOXK1 | 886.161881791868 | 1.22316861362982 | 0.104970352581313 | 11.6525150535464 | 2.22785017775319e-31 | 8.0032865433191e-30 | UP |
| PFKP | 825.468855862126 | 1.50968844332237 | 0.256629962220517 | 5.88274428386941 | 4.0351912462354e-09 | 1.63102672318902e-08 | UP |
| ARL2 | 901.719266020523 | 1.01305464299538 | 0.15069747404247 | 6.72243943989321 | 1.7870702221567e-11 | 9.64905364725893e-11 | UP |
| TKTL1 | 22.1068787509038 | 1.52868939262095 | 0.399825171726214 | 3.82339457523636 | 0.000131626885690042 | 0.000298554788227803 | UP |

**Supplementary Table 7.** The C-index of the risk model.

| Model | C_index | SE | 95%_CI |
| --- | --- | --- | --- |
| Training set | 0.627 | 0.0224 | （0.583, 0.671） |
| Validation set | 0.644 | 0.0354 | （0.574, 0.713） |

**Supplementary Table 8.** Differential immune checkpoints between high and low-risk groups.

| gene | .y. | group1 | group2 | n1 | n2 | statistic | p | p.adj |
| --- | --- | --- | --- | --- | --- | --- | --- | --- |
| BTLA | expr | High | Low | 184 | 184 | 19221.5 | 0.0246 | 0.0319135135135135 |
| BTNL2 | expr | High | Low | 184 | 184 | 18477 | 0.0328 | 0.0414315789473684 |
| C10orf54 | expr | High | Low | 184 | 184 | 22003 | 6.58e-07 | 1.504e-06 |
| CD200 | expr | High | Low | 184 | 184 | 21686 | 3.12e-06 | 5.9904e-06 |
| CD200R1 | expr | High | Low | 184 | 184 | 21228 | 2.51e-05 | 4.30285714285714e-05 |
| CD27 | expr | High | Low | 184 | 184 | 20990 | 6.87e-05 | 0.000113710344827586 |
| CD274 | expr | High | Low | 184 | 184 | 19338 | 0.0182 | 0.02496 |
| CD276 | expr | High | Low | 184 | 184 | 24916 | 4.94e-15 | 1.1856e-13 |
| CD28 | expr | High | Low | 184 | 184 | 20090 | 0.00194 | 0.00291 |
| CD40 | expr | High | Low | 184 | 184 | 20018 | 0.00246 | 0.00357818181818182 |
| CD44 | expr | High | Low | 184 | 184 | 21248 | 2.3e-05 | 4.08888888888889e-05 |
| CD48 | expr | High | Low | 184 | 184 | 20279 | 0.00102 | 0.00157935483870968 |
| CD70 | expr | High | Low | 184 | 184 | 22174 | 2.72e-07 | 7.25333333333333e-07 |
| CD80 | expr | High | Low | 184 | 184 | 23243 | 6.06e-10 | 2.424e-09 |
| CD86 | expr | High | Low | 184 | 184 | 22969 | 3.22e-09 | 1.18892307692308e-08 |
| CTLA4 | expr | High | Low | 184 | 184 | 22484.5 | 5.17e-08 | 1.6544e-07 |
| HAVCR2 | expr | High | Low | 184 | 184 | 23646 | 4.59e-11 | 4.216e-10 |
| HHLA2 | expr | High | Low | 184 | 184 | 23150 | 3.37e-10 | 1.62763636363636e-09 |
| ICOS | expr | High | Low | 184 | 184 | 22045.5 | 5.3e-07 | 1.33894736842105e-06 |
| IDO2 | expr | High | Low | 184 | 184 | 14101 | 0.00559 | 0.00789176470588235 |
| LAG3 | expr | High | Low | 184 | 184 | 19230 | 0.0241 | 0.0319135135135135 |
| LAIR1 | expr | High | Low | 184 | 184 | 23950 | 5.92e-12 | 7.104e-11 |
| LGALS9 | expr | High | Low | 184 | 184 | 24975 | 3.12e-15 | 1.1856e-13 |
| NRP1 | expr | High | Low | 184 | 184 | 20503 | 0.00046 | 0.000736 |
| PDCD1 | expr | High | Low | 184 | 184 | 21589 | 4.93e-06 | 9.10153846153846e-06 |
| PDCD1LG2 | expr | High | Low | 184 | 184 | 19048 | 0.0378 | 0.0465230769230769 |
| SELPLG | expr | High | Low | 184 | 184 | 21866 | 1.3e-06 | 2.71304347826087e-06 |
| TIGIT | expr | High | Low | 184 | 184 | 21708 | 2.81e-06 | 5.62e-06 |
| TNFRSF14 | expr | High | Low | 184 | 184 | 23510 | 1.12e-10 | 7.68e-10 |
| TNFRSF18 | expr | High | Low | 184 | 184 | 24254 | 6.99e-13 | 1.1184e-11 |
| TNFRSF25 | expr | High | Low | 184 | 184 | 22343 | 1.12e-07 | 3.16235294117647e-07 |
| TNFRSF4 | expr | High | Low | 184 | 184 | 23472 | 1.43e-10 | 8.58e-10 |
| TNFRSF8 | expr | High | Low | 184 | 184 | 22456 | 6.05e-08 | 1.815e-07 |
| TNFRSF9 | expr | High | Low | 184 | 184 | 22035 | 5.59e-07 | 1.3416e-06 |
| TNFSF15 | expr | High | Low | 184 | 184 | 23625 | 5.27e-11 | 4.216e-10 |
| TNFSF18 | expr | High | Low | 184 | 184 | 21884 | 1.19e-06 | 2.59636363636364e-06 |
| TNFSF4 | expr | High | Low | 184 | 184 | 22666 | 1.87e-08 | 6.41142857142857e-08 |
| TNFSF9 | expr | High | Low | 184 | 184 | 23331 | 3.5e-10 | 1.62763636363636e-09 |
| VTCN1 | expr | High | Low | 184 | 184 | 23297 | 3.73e-10 | 1.62763636363636e-09 |

**Supplementary Table 9.** Drugs with significant differences in IC50 values between high-risk and low-risk groups.

| high_group | low_group | padj | pvalue | log2FoldChange | drugs | sig |
| --- | --- | --- | --- | --- | --- | --- |
| -0.5721 | -0.6451 | 1.48E-29 | 1.72E-30 | 0.081725994 | A.443654 | A.443654**** |
| 3.8 | 4.04 | 1.83E-16 | 7.31E-17 | -0.212583429 | A.770041 | A.770041**** |
| 3.144 | 2.422 | 2.43E-30 | 2.64E-31 | 0.662361175 | ABT.263 | ABT.263**** |
| 5.375 | 5.291 | 8.76E-07 | 6.22E-07 | 0.055476362 | ABT.888 | ABT.888**** |
| 4.154 | 4.196 | 0.001469018 | 0.001160312 | -0.068451242 | AG.014699 | AG.014699** |
| 8.114 | 8.113 | 0.007101117 | 0.006020512 | 0.000562711 | AICAR | AICAR** |
| 4.29 | 4.192 | 3.21E-05 | 2.37E-05 | 0.059998432 | AMG.706 | AMG.706**** |
| 1.594 | 1.71 | 1.19E-16 | 4.67E-17 | -0.138991973 | AP.24534 | AP.24534**** |
| 3.144 | 3.217 | 3.82E-15 | 1.63E-15 | -0.065472615 | AS601245 | AS601245**** |
| 5.352 | 5.067 | 9.75E-29 | 1.27E-29 | 0.262366215 | ATRA | ATRA**** |
| -2.74 | -2.607 | 7.85E-22 | 2.11E-22 | -0.153296235 | AUY922 | AUY922**** |
| 3.425 | 3.339 | 1.40E-10 | 8.01E-11 | 0.066397881 | Axitinib | Axitinib**** |
| 3.068 | 3.228 | 8.70E-18 | 3.22E-18 | -0.17694749 | AZ628 | AZ628**** |
| 3.984 | 4.32 | 5.76E-22 | 1.50E-22 | -0.283818702 | AZD.0530 | AZD.0530**** |
| 4.899 | 4.75 | 0.003708383 | 0.003063447 | 0.081749006 | AZD.2281 | AZD.2281** |
| 2.983 | 3.073 | 8.26E-10 | 4.97E-10 | -0.121887679 | AZD6244 | AZD6244**** |
| 3.447 | 3.448 | 8.20E-19 | 2.73E-19 | -0.001255173 | AZD6482 | AZD6482**** |
| 0.3459 | 0.3493 | 0.010217387 | 0.008736607 | -0.005958642 | AZD8055 | AZD8055** |
| 2.481 | 2.333 | 2.30E-07 | 1.60E-07 | 0.104147602 | BAY.61.3606 | BAY.61.3606**** |
| 4.698 | 4.864 | 1.74E-19 | 5.42E-20 | -0.171294745 | Bexarotene | Bexarotene**** |
| -1.59 | -1.591 | 1.25E-07 | 8.25E-08 | 0.000969244 | BI.2536 | BI.2536**** |
| 4.473 | 4.565 | 9.39E-14 | 4.76E-14 | -0.071808095 | Bicalutamide | Bicalutamide**** |
| 5.892 | 5.884 | 9.89E-07 | 7.10E-07 | 0.006579083 | BIRB.0796 | BIRB.0796**** |
| 1.554 | 1.694 | 1.20E-33 | 6.11E-35 | -0.146154258 | Bleomycin | Bleomycin**** |
| 3.693 | 3.83 | 3.87E-16 | 1.60E-16 | -0.165660665 | BMS.509744 | BMS.509744**** |
| 2.362 | 2.925 | 2.66E-25 | 4.82E-26 | -0.518093113 | BMS.536924 | BMS.536924**** |
| 4.948 | 4.94 | 8.12E-07 | 5.71E-07 | 0.007493318 | BMS.708163 | BMS.708163**** |
| 1.059 | 1.437 | 2.35E-30 | 2.38E-31 | -0.356431356 | BMS.754807 | BMS.754807**** |
| -5.314 | -5.03 | 7.35E-17 | 2.82E-17 | -0.240097278 | Bortezomib | Bortezomib**** |
| 2.751 | 2.787 | 0.003321059 | 0.002695352 | -0.046980038 | Bosutinib | Bosutinib** |
| -1.23 | -1.065 | 4.19E-34 | 1.82E-35 | -0.16125585 | Bryostatin.1 | Bryostatin.1**** |
| -3.995 | -4.14 | 0.049891772 | 0.044468754 | 0.056161381 | Camptothecin | Camptothecin* |
| 5.626 | 5.577 | 7.49E-24 | 1.74E-24 | 0.043969372 | CCT007093 | CCT007093**** |
| 3.17 | 3.184 | 5.03E-31 | 4.01E-32 | -0.0170343 | CCT018159 | CCT018159**** |
| 4.063 | 4.046 | 1.30E-32 | 8.47E-34 | 0.018660008 | CGP.082996 | CGP.082996**** |
| -1.767 | -1.636 | 3.68E-18 | 1.28E-18 | -0.154356372 | CGP.60474 | CGP.60474**** |
| 4.736 | 4.926 | 7.03E-37 | 5.09E-39 | -0.194651282 | CHIR.99021 | CHIR.99021**** |
| 2.545 | 2.728 | 1.20E-11 | 6.52E-12 | -0.192790217 | CI.1040 | CI.1040**** |
| 3.379 | 3.43 | 2.22E-05 | 1.63E-05 | -0.059377253 | Cisplatin | Cisplatin**** |
| 3.659 | 3.67 | 2.04E-05 | 1.47E-05 | -0.008971789 | CMK | CMK**** |
| 6.521 | 6.451 | 3.61E-14 | 1.70E-14 | 0.057505974 | Cyclopamine | Cyclopamine**** |
| 0.6644 | 0.7208 | 0.000144088 | 0.000110676 | -0.086410473 | Cytarabine | Cytarabine*** |
| 1.836 | 2.724 | 5.59E-24 | 1.26E-24 | -0.807051069 | Dasatinib | Dasatinib**** |
| 6.625 | 6.54 | 1.78E-11 | 9.82E-12 | 0.067320549 | DMOG | DMOG**** |
| -5.483 | -5.205 | 6.03E-31 | 5.24E-32 | -0.269411709 | Docetaxel | Docetaxel**** |
| 4.729 | 4.577 | 3.35E-16 | 1.36E-16 | 0.12578564 | EHT.1864 | EHT.1864**** |
| -2.964 | -2.811 | 2.27E-07 | 1.56E-07 | -0.15865615 | Elesclomol | Elesclomol**** |
| 2.872 | 2.879 | 4.00E-25 | 7.54E-26 | -0.00708961 | Embelin | Embelin**** |
| -5.142 | -4.894 | 6.51E-21 | 1.89E-21 | -0.216561503 | Epothilone.B | Epothilone.B**** |
| 4.469 | 4.72 | 5.87E-14 | 2.89E-14 | -0.202248649 | Erlotinib | Erlotinib**** |
| 1.621 | 1.576 | 7.95E-26 | 1.38E-26 | 0.040875477 | Etoposide | Etoposide**** |
| 2.099 | 2.183 | 1.50E-08 | 9.70E-09 | -0.065225442 | FH535 | FH535**** |
| 3.617 | 3.858 | 8.02E-36 | 1.29E-37 | -0.247091313 | FTI.277 | FTI.277**** |
| 5.736 | 5.725 | 0.0409236 | 0.036178835 | 0.006814594 | GDC.0449 | GDC.0449* |
| 2.187 | 2.223 | 0.027374817 | 0.02360582 | -0.044477549 | GDC0941 | GDC0941* |
| 2.017 | 2.017 | 0.000135296 | 0.000102942 | 3.45E-05 | Gefitinib | Gefitinib*** |
| -2.451 | -2.381 | 0.002013144 | 0.001619268 | -0.128426434 | Gemcitabine | Gemcitabine** |
| 4.504 | 4.691 | 1.12E-27 | 1.78E-28 | -0.172188274 | GNF.2 | GNF.2**** |
| 3.605 | 3.801 | 5.97E-14 | 2.98E-14 | -0.169626279 | GSK269962A | GSK269962A**** |
| 4.093 | 4.146 | 1.80E-07 | 1.22E-07 | -0.073380409 | GSK.650394 | GSK.650394**** |
| 4.256 | 4.324 | 8.46E-16 | 3.56E-16 | -0.056489841 | GW.441756 | GW.441756**** |
| -2.311 | -2.356 | 5.18E-20 | 1.58E-20 | 0.042480703 | GW843682X | GW843682X**** |
| 4.974 | 5.036 | 8.81E-25 | 1.92E-25 | -0.073238745 | Imatinib | Imatinib**** |
| 5.937 | 5.6 | 7.47E-18 | 2.71E-18 | 0.269563285 | IPA.3 | IPA.3**** |
| 2.864 | 2.968 | 1.02E-18 | 3.48E-19 | -0.113752467 | JNJ.26854165 | JNJ.26854165**** |
| -0.2643 | -0.2367 | 4.85E-18 | 1.72E-18 | -0.028318641 | JNK.9L | JNK.9L**** |
| 5.889 | 5.868 | 0.006745663 | 0.005670267 | 0.014641474 | JNK.Inhibitor.VIII | JNK.Inhibitor.VIII** |
| -1.626 | -1.627 | 0.001931494 | 0.001539596 | 0.00231693 | JW.7.52.1 | JW.7.52.1** |
| 6.096 | 6.145 | 8.28E-11 | 4.68E-11 | -0.053453049 | KIN001.135 | KIN001.135**** |
| 5.07 | 5.182 | 3.84E-14 | 1.83E-14 | -0.104073958 | KU.55933 | KU.55933**** |
| 4.288 | 4.491 | 7.45E-05 | 5.61E-05 | -0.169419758 | Lapatinib | Lapatinib**** |
| 5.411 | 5.355 | 1.32E-13 | 6.77E-14 | 0.047648373 | Lenalidomide | Lenalidomide**** |
| 6.096 | 6.233 | 8.02E-36 | 2.33E-37 | -0.141669842 | LFM.A13 | LFM.A13**** |
| 10.57 | 10.39 | 6.21E-28 | 9.45E-29 | 0.176778078 | Metformin | Metformin**** |
| 1.125 | 0.7514 | 3.19E-17 | 1.20E-17 | 0.301611922 | Methotrexate | Methotrexate**** |
| 1.478 | 1.848 | 2.06E-19 | 6.55E-20 | -0.388655096 | MG.132 | MG.132**** |
| 0.4371 | 0.8454 | 5.66E-35 | 2.05E-36 | -0.405624334 | Midostaurin | Midostaurin**** |
| -0.9916 | -1.029 | 4.77E-25 | 9.68E-26 | 0.042328691 | Mitomycin.C | Mitomycin.C**** |
| 2.857 | 2.692 | 2.02E-11 | 1.13E-11 | 0.152324458 | MK.2206 | MK.2206**** |
| 0.8865 | 0.7238 | 9.75E-29 | 1.24E-29 | 0.143340633 | MS.275 | MS.275**** |
| 4.296 | 4.177 | 5.58E-09 | 3.48E-09 | 0.096872749 | Nilotinib | Nilotinib**** |
| 6.739 | 6.849 | 2.54E-19 | 8.29E-20 | -0.100015333 | NSC.87877 | NSC.87877**** |
| 3.447 | 3.583 | 3.41E-08 | 2.22E-08 | -0.145418519 | NU.7441 | NU.7441**** |
| 4.848 | 4.625 | 7.82E-09 | 4.99E-09 | 0.172221524 | Nutlin.3a | Nutlin.3a**** |
| -2.515 | -2.25 | 7.68E-15 | 3.45E-15 | -0.264351868 | NVP.BEZ235 | NVP.BEZ235**** |
| 1.644 | 1.823 | 1.55E-20 | 4.60E-21 | -0.160148185 | NVP.TAE684 | NVP.TAE684**** |
| -1.019 | -1.042 | 0.00015159 | 0.000117537 | 0.017867985 | Obatoclax.Mesylate | Obatoclax.Mesylate*** |
| 3.68 | 3.702 | 8.02E-36 | 1.76E-37 | -0.023775243 | OSI.906 | OSI.906**** |
| 3.705 | 3.402 | 3.03E-28 | 4.18E-29 | 0.283617449 | PAC.1 | PAC.1**** |
| -2.873 | -3.027 | 0.003487471 | 0.002855682 | 0.157611053 | Paclitaxel | Paclitaxel** |
| 4.89 | 4.978 | 1.21E-09 | 7.44E-10 | -0.127533944 | Parthenolide | Parthenolide**** |
| 4.466 | 4.575 | 6.21E-28 | 9.24E-29 | -0.124758485 | Pazopanib | Pazopanib**** |
| 0.07493 | 0.403 | 7.11E-32 | 5.15E-33 | -0.320251332 | PD.0325901 | PD.0325901**** |
| 4.152 | 4.004 | 2.78E-22 | 7.04E-23 | 0.12715924 | PD.173074 | PD.173074**** |
| 4.387 | 4.355 | 1.43E-07 | 9.66E-08 | 0.024294288 | PF.02341066 | PF.02341066**** |
| 4.96 | 4.941 | 3.48E-05 | 2.60E-05 | 0.030884516 | PF.4708671 | PF.4708671**** |
| 2.702 | 2.793 | 4.89E-21 | 1.38E-21 | -0.079952726 | PF.562271 | PF.562271**** |
| 4.919 | 4.987 | 3.55E-12 | 1.90E-12 | -0.052835505 | PHA.665752 | PHA.665752**** |
| 4.951 | 4.864 | 0.02965059 | 0.025997981 | 0.035917378 | PLX4720 | PLX4720* |
| 4.477 | 4.633 | 7.27E-15 | 3.22E-15 | -0.148908055 | Pyrimethamine | Pyrimethamine**** |
| -0.1901 | -0.2871 | 1.17E-30 | 1.10E-31 | 0.096713471 | Rapamycin | Rapamycin**** |
| 2.103 | 2.693 | 1.01E-32 | 5.84E-34 | -0.570606723 | RDEA119 | RDEA119**** |
| 4.668 | 4.702 | 5.61E-09 | 3.54E-09 | -0.037773653 | RO.3306 | RO.3306**** |
| 4.185 | 4.044 | 1.24E-26 | 2.06E-27 | 0.143577005 | Salubrinal | Salubrinal**** |
| 5.55 | 5.605 | 1.11E-09 | 6.74E-10 | -0.079615377 | SB.216763 | SB.216763**** |
| 5.075 | 4.883 | 4.51E-10 | 2.65E-10 | 0.152360058 | SB590885 | SB590885**** |
| 5.621 | 5.593 | 0.029597176 | 0.025736675 | 0.009742316 | SL.0101.1 | SL.0101.1* |
| 1.57 | 1.568 | 1.39E-07 | 9.25E-08 | 0.001915003 | S.Trityl.L.cysteine | S.Trityl.L.cysteine**** |
| 3.314 | 3.293 | 1.11E-14 | 5.06E-15 | 0.017778664 | Sunitinib | Sunitinib**** |
| -4.367 | -4.077 | 4.65E-25 | 9.09E-26 | -0.267909597 | Thapsigargin | Thapsigargin**** |
| 2.114 | 2.193 | 4.83E-13 | 2.52E-13 | -0.096670881 | Tipifarnib | Tipifarnib**** |
| -3.886 | -3.912 | 0.000266809 | 0.000208807 | 0.031315946 | Vinorelbine | Vinorelbine*** |
| 1.396 | 1.049 | 6.42E-15 | 2.79E-15 | 0.26701627 | Vorinostat | Vorinostat**** |
| 2.665 | 2.654 | 1.41E-22 | 3.48E-23 | 0.01132462 | VX.680 | VX.680**** |
| 4.415 | 4.265 | 5.74E-10 | 3.41E-10 | 0.110031522 | VX.702 | VX.702**** |
| 3.884 | 4.331 | 3.10E-14 | 1.44E-14 | -0.377247202 | WH.4.023 | WH.4.023**** |
| 2.872 | 3.157 | 2.51E-21 | 6.91E-22 | -0.243464336 | WO2009093972 | WO2009093972**** |
| 5.624 | 5.686 | 0.004969275 | 0.004141063 | -0.043030465 | WZ.1.84 | WZ.1.84** |
| -1.002 | -0.4294 | 3.44E-23 | 8.22E-24 | -0.459990816 | X17.AAG | X17.AAG**** |
| 2.507 | 2.352 | 3.08E-10 | 1.78E-10 | 0.119383915 | X681640 | X681640**** |
| 3.278 | 3.24 | 7.19E-25 | 1.51E-25 | 0.036138818 | XMD8.85 | XMD8.85**** |
| 2.574 | 2.689 | 5.89E-13 | 3.12E-13 | -0.112035104 | Z.LLNle.CHO | Z.LLNle.CHO**** |
| 2.822 | 2.63 | 5.08E-14 | 2.47E-14 | 0.152745615 | ZM.447439 | ZM.447439**** |
